# Recombinant hTRBP and hPACT Modulate hAgo2-Catalyzed siRNA-Mediated Target RNA Cleavage In Vitro

**DOI:** 10.1371/journal.pone.0146814

**Published:** 2016-01-19

**Authors:** Sarah Willkomm, Andrea Deerberg, Johannes Heidemann, Friedemann Flügge, Janica Meine, Rui Hu, Rosel Kretschmer-Kazemi Far, Tobias Restle

**Affiliations:** 1 Institute of Microbiology, Single Molecule Biochemistry Lab, University of Regensburg, Regensburg, 93053, Germany; 2 Institute of Molecular Medicine, Universitätsklinikum Schleswig-Holstein, University of Lübeck, Lübeck, 23538, Germany; Institute of Molecular Genetics IMG-CNR, ITALY

## Abstract

The human TAR RNA-binding protein (hTRBP) and protein activator of protein kinase R (hPACT) are important players in RNA interference (RNAi). Together with hArgonaute2 (hAgo2) and hDicer they have been reported to form the RISC-loading complex (RLC). Among other functions, hTRBP was suggested to assist the loading of hAgo2 with small interfering RNAs (siRNAs) within the RLC. Although several studies have been conducted to evaluate the specific functions of hTRBP and hPACT in RNAi, exact mechanisms and modes of action are still unknown. Here, we present a biochemical study further evaluating the role of hTRBP and hPACT in hAgo2-loading. We found that both proteins enhance hAgo2-mediated RNA cleavage significantly; even a hAgo2 mutant impaired in siRNA binding shows full cleavage activity in the presence of hTRBP or hPACT. Pre-steady state binding studies reveal that the assembly of wildtype-hAgo2 (wt-hAgo2) and siRNAs remains largely unaffected, whereas the binding of mutant hAgo2-PAZ9 to siRNA is restored by adding either hTRBP or hPACT. We conclude that both proteins assist in positioning the siRNA within hAgo2 to ensure optimal binding and cleavage. Overall, our data indicate that hTRBP and hPACT are part of a regulative system of RNAi that is important for efficient target RNA cleavage.

## Introduction

RNA interference (RNAi) [1] is an evolutionarily conserved posttranscriptional process that leads to sequence-specific regulation of gene expression. It is mediated by small interfering RNAs (siRNAs) of 21–23 nucleotides in length that are generated from long double-stranded RNAs (dsRNAs) [2,3] by the RNase III Dicer [4,5]. siRNAs are incorporated into the RNA-silencing complex (RISC) [3,6] by the RISC-loading complex (RLC). RLC is composed of Argonaute2 (Ago2), Dicer, siRNA and trans-activation response (TAR) RNA-binding protein (TRBP) or protein activator of protein kinase R (PKR) (PACT). Within the RLC, the siRNA is loaded into Ago2 which is the catalytically active component of RISC [7–10]. Subsequently, one of the strands of the siRNA is removed and the remaining strand is used as a guide strand to mediate cleavage of complementary target RNAs [11].

While TRBP and PACT were identified as part of the RLC almost 10 years ago, their exact function in siRNA-guided RNA cleavage remains elusive. TRBP is a dsRNA-binding protein that was first identified as a protein that binds TAR RNA [12]. Later, TRBP was described as an inhibitor of PKR [13,14] and was found to play a role in RNAi [15,16]. As a member of the large dsRNA-binding protein family, TRBP contains three evolutionary conserved domains called dsRNA-binding domains (dsRBDs). The first two dsRBDs are able to bind the A-form helix of dsRNAs in a sequence-independent manner, whereas the C-terminal dsRBD does not have corresponding residues for RNA binding [17] and therefore does not bind dsRNA [18,19]. The C-terminal dsRBD is called Medipal domain and is involved in interactions with proteins like Dicer and PACT [15,20]. TRBP is able to form homodimers via the dsRBD1 & 2 [20].

PACT is also a dsRNA-binding protein and was first identified as an activator of PKR [21]. Later, it was described to be part of the RLC [7,22]. As described for TRBP, PACT consists of three dsRBDs. Likewise, the first two domains are important for binding to RNA and PKR, while the third domain plays a role in PKR-activation [23]. Besides being part of the RLC, PACT could be shown to directly interact with Dicer and Ago2 [22,24]. Moreover, PACT seems to be involved in guide strand selection [25]. PACT is able to form homodimers as well as heterodimers with TRBP via its third domain [24,26].

Even though opposite functions of PACT and TRBP were described in PKR, their roles in RNAi seem to be rather related. However, the effect of PACT in comparison to TRBP is less pronounced [22].

In the present biochemical study we further specified the role of hTRBP and hPACT in RNAi. We analyzed the thermodynamics and transient kinetics of complex formation between the dsRNA-binding proteins and siRNA, their effect on hAgo2/siRNA association, hAgo2-mediated target RNA cleavage and product dissociation. We found that hTRBP, as well as a C-terminal deletion mutant (hTRBP-D12), and hPACT stimulate hAgo2-mediated cleavage of RNAs. Moreover, hTRBP and hPACT restored binding of siRNA to the binding-impaired hAgo2-PAZ9 mutant, which led to a rescue of cleavage activity. Our results imply hTRBP and hPACT are part of a regulative system that modulate the efficiency of RNAi.

## Results

### Kinetic analysis of the assembly of siRNA with hTRBP or hPACT reveals two distinct phases

**Fig 1:**
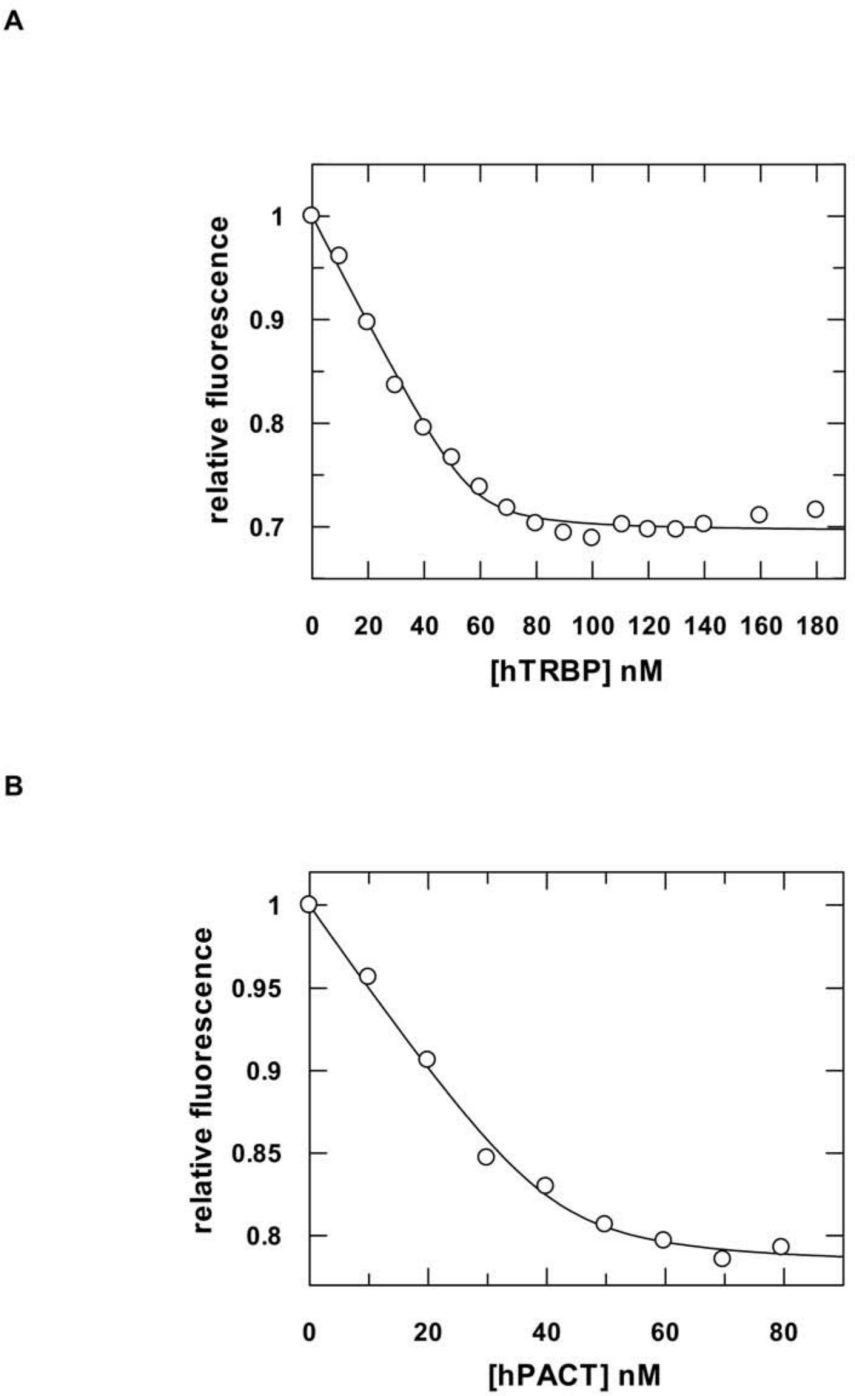
Equilibrium titrations of hTRBP and hPACT with double-stranded siRNA. Representative binding experiments are show. 50 nM fluorescently labeled double-stranded siRNA (aslam-FAM/slam) were titrated with increasing concentrations of hTRBP (A) and hPACT (B). Experimental data were fit to a quadratic equation which yields *K*_*d*_ values of 1.2 (± 0.4) nM and 1.7 (± 0.5) nM for hTRBP and hPACT, respectively. Averaging four individual measurements each we derived *K*_d's_ of 3.4 (± 1.6) and 2.8 (± 1.8) nM for hTRBP and hPACT, respectively; numbers in brackets represent standard deviation.

Initially, we performed nucleic acid binding experiments with either hTRBP or hPACT purified under denaturing conditions. Both proteins were subsequently refolded as described in the Methods section. Steady state binding experiments were performed with a fluorescence spectrometer using a siRNA substrate (aslam-FAM/slam) carrying a 5’-FAM label termed silam-FAM (Table 1). Equilibrium titrations yielded a *K*_d_ of 3.1 nM for hTRBP^denat^ and 2 nM for hPACT^denat^ (Table 2 and Figure B in S1 Supporting Information File). For reasons explained below, we eventually decided to purify the two proteins under native conditions. Here, equilibrium titrations yielded a comparable *K*_d_ of 3.4 nM for hTRBP and 2.8 nM for hPACT (Fig 1 and Table 2), respectively.

**Table 1:** Sequences of guide and target RNAs and in vitro transcripts used in the present study.

RNA	Sequence
**as2b**	5´-UAGAGGUACGUGCUGAGGCdTdT-3´
**as2b-FAM**	5´-UAGAGGUACGUGCT*GAGGCdTdT-3´
**s2b**	5´-GCCUCAGC**AC**GUACCUCUAdTdT-3´
**ICAM-1-IVT**	5’-GGGCGAAUUGGGCCCGACGUCGCAUGCUCCCGGCCGCCAUGGCCG CGGGAUUAGCCGCAGUCAUAAUGGGCACUGCAGGCCUCAGC**AC**GUACC UCUAUAACCGCCAGCGGAAGAUCAAGAAAUAAAUCACUAGUGCGGCC-3’
**aslam**	5’-UGUUCUUCUGGAAGUCCAGdTdT-3’
**aslam-FAM**	5’-*UGUUCUUCUGGAAGUCCAGdTdT-3’
**slam**	5’- CUGGACUUCCAGAAGAACAdTdT-3’

position of FAM-label, cleavage position (s2b and ICAM-1-IVT) is indicated by bold letters, the guide matching sequence in ICAM-1-IVT is underlined.

**Table 2:** Summary of equilibrium and pre-steady state binding data for complex formation between TRBP^nat or denat^ or PACT^nat or denat^ and siRNA.

	*K*_d_meas_ (nM)	*k*_1_ (M^-1^s^-1^)	*k*_-1_ (s^-1^)	*k*_2_ (s^-1^)	*k*_*-2*_ (s^-1^)	*K*_d_cal_ (nM)
**hTRBP**^**denat**^	3.1 (± 0.5)	2.0 (± 0.04) x 10^8^	3.2 (± 0.03)	2.7 (± 0.6)	0.15 (± 0.004)	0.8
**hTRBP**^**nat**^	3.4 (± 1.6)	0.1 (± 0.06) x 10^8^	0.15 (± 0.04)	0.03 (± 0.003)	0.03 (± 0.008)	15
**hPACT**^**denat**^	2 (± 0.6)	1.9 (± 0.04) x 10^8^	0.8 (± 0.07)	1.7 (± 0.1)	0.17 (± 0.05)	0.3
**hPACT**^**nat**^	2.8 (± 1.8)	0.5 (± 0.04) x 10^8^	n.d.	0.01 (± 0.007)	n.d.	n.d.

Equilibrium measurements are represented by *K*_d_meas_, while *K*_d_cal_ is calculated from corresponding association and dissociation rate constants (*K*_d_cal_ = *k*_off_/*k*_on_ = (*k*_-1_/*k*_1_)x(*k*_-2_/*k*_2_)x(*k*_-3_/*k*_3_)). Rate constants are averaged from up to five independent measurements. Standard deviations in brackets. For *k*_1_ standard errors are given.

**Fig 2:**
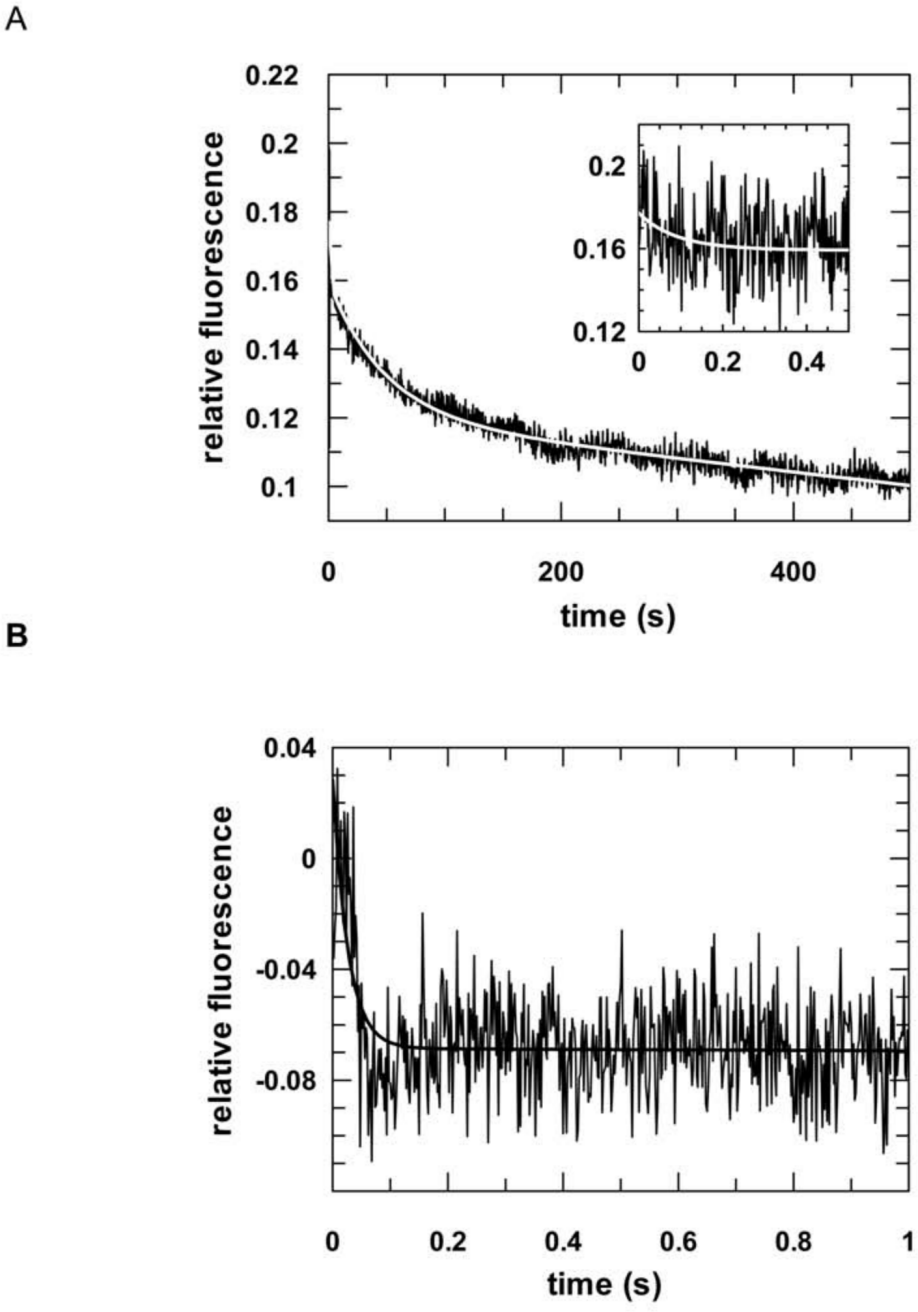
Pre-steady state kinetic analyses of the association of hTRBP or hPACT and double-stranded siRNA. Representative stopped-flow graphs are shown. The inserts show the reaction on a shorter time scale. 50 nM fluorescently labeled double-stranded siRNA (aslam-FAM/slam) were rapidly mixed with 500 nM hTRBP (A) or hPACT (B). Data were fitted to a double exponential equation yielding the following rates: *k*_*1*_: 11.1 (± 2.3) s^-1^ and *k*_*2*_: 0.02 (± 0.001) s^-1^ for hTRBP and *k*_*1*_: 37.8 (± 2.5) s^-1^ and *k*_*2*_: 0.04 (± 0.005) s^-1^ for hPACT.

Next, we analyzed complex formation of hTRBP or hPACT and siRNA under pre-steady state conditions using a stopped-flow apparatus with the same components as described above. Data obtained for both proteins, regardless if purified under denaturing or native conditions, were best fitted to an equation with two exponential terms (Fig 2, Figure C in S1 Supporting Information File and Table 2). For both proteins the first phase is fast and dependent on the protein concentration (Figures D and E in S1 Supporting Information File), whereas the second phase is not. This implies that the first phase represents a diffusion-controlled process, i.e. the formation of a collision complex. For the second order rate constants *k*_1_, we determined values of 2.0 x 10^8^ (± 0.04 x 10^8^) M^-1^ s^-1^ and 1.9 x 10^8^ (± 0.04 x 10^8^) M^-1^ s^-1^ for hTRBP^denat^ and hPACT^denat^, and 0.1 x 10^8^ (± 0.06 x 10^8^) M^-1^ s^-1^ and 0.5 x 10^8^ (± 0.04 x 10^8^) M^-1^ s^-1^ for hTRBP^nat^ and hPACT^nat^, respectively. Interestingly, complex formation is up to 10-fold faster in case of protein purified under denaturing conditions. This finding was even more pronounced in case of the second association rate constant. Here, we observed differences of up to 170-fold. Dissociation was analyzed using a pre-assembled complex comprising hTRBP or hPACT and fluorescently labelled siRNA that was competed with an excess of unlabelled siRNA. Again, data could be best fitted to an equation with two exponential terms (Figures F and G in S1 Supporting Information File). Likewise, we observed significant differences regarding the observed dissociation rate constants with complexes comprised of native purified protein falling apart up to 20-fold slower. In case of hPACT^nat^, determination of dissociation rate constants turned out to be impracticable.

Interestingly, while the determined equilibrium dissociation constants of all four proteins (TRBP^nat or denat^ and PACT^nat or denat^) are about the same, the association and dissociation rate constants are quite different dependent on the particular purification conditions (Table 2). The reduced second order rate constants in the case of protein purified under native conditions are most likely due to protein aggregation negatively affecting molecule diffusion. This interpretation would also explain the overestimated calculated *K*_d_ value of 15 nM in case of hTRBP^nat^. On the other hand, the differences in the remaining transient rate constants clearly indicate different conformational transitions upon binary complex formation between protein and RNA.

### hTRBP and hPACT enhance passenger cleavage and siRNA-mediated target RNA cleavage

In order to investigate if dsRNA-binding proteins exert a functional role in RNAi, we performed cleavage assays to examine potential effects of hTRBP and hPACT on hAgo2-mediated target RNA cleavage. Initially, experiments were performed with either hTRBP or hPACT purified under denaturing conditions as described above. In neither case we observed any effects. Astonishingly, after reconsidering our purification protocol and switching to the native purification procedure, the picture changed entirely. Consequently, all further experiments described in the present study were performed using protein purified under native conditions.

**Fig 3:**
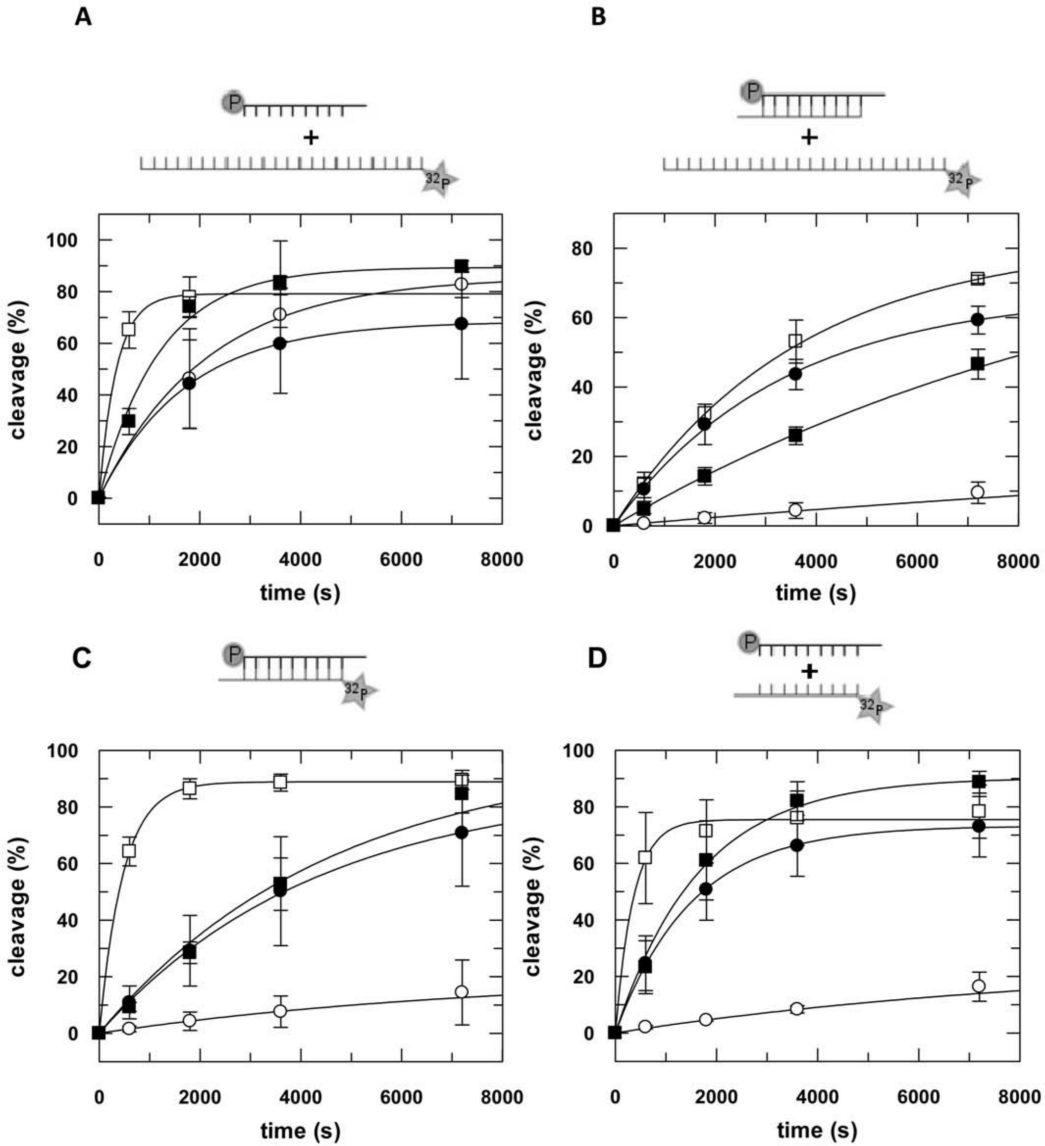
Effect of dsRNA-binding proteins on hAgo2-catalyzed target RNA cleavage as a function of different substrates. The different substrate combinations (ss-siRNA & long target, ds-siRNA & long target, ds-siRNA or ss-siRNA & short target) are depicted as little cartoons on top of the corresponding graph. For target RNA cleavage either 2.5 nM radioactively labeled ICAM-1-IVT (A, B) or s2b (D) were mixed with binary complexes consisting of either 100 nM as2b (A, D) or as2b/s2b (B) and 3 μM hAgo2 in the absence (open circles) or presence of 3 μM hTRBP (closed circles), hTRBP-D12 (open squares) and hPACT (closed squares), respectively. For ds-siRNA cleavage, i.e. passenger cleavage (C), 30 nM 5’-32P-passenger labeled siRNA (as2b/s2b) was mixed with 3 μM hAgo2 in the presence or absence of dsRBPs as described above. Samples were taken at different time points, analyzed by denaturing PAGE (8% for ICAM-1-IVT and 20% for s2b) and detected by autoradiography (Figure H in S1 Supporting Information File). Data shown are averaged from at least three independent measurements. Error bars represent standard deviation. Experimental data were fitted to an exponential equation. Rate constants and corresponding amplitudes are listed in Table 3.

Cleavage experiments were performed by assembling a binary complex of hAgo2 and single- or double-stranded siRNA (as2b or as2b/s2b, Table 1) in the absence or presence of hTRBP, hTRBP-D12 or hPACT in cleavage buffer (Table 3). The reaction was started by adding a guide-matching 140 nt radio-labelled target RNA ICAM-1-IVT (Fig 3A and 3B). The observed cleavage rate remained essentially unaffected by the presence of the full-length dsRNA-binding proteins (dsRBPs). In case of a C-terminal deletion mutant hTRBP-D12 (i.e. the third dsRNA-binding domain is missing), when hAgo2 was incubated with single-stranded siRNA, we measured an about 10-fold faster cleavage rate. Remarkably, in case of double-stranded siRNA, we observed a massive boost of cleavage products in the presence of all three dsRBPs. The amount of cleavage product increased from 18% to values between 67% and 95% (Table 3). This was not the case when hAgo2 was programmed with a single-stranded siRNA. Here, the level of cleavage was comparable to the reaction without dsRBPs for hTRBP-D12 and hPACT, while hTRBP even caused a significant decrease. Moreover, we investigated the effect of dsRBPs on passenger and short 21 nt target RNA cleavage as compared to the 140 nt long target described above (Fig 3C and 3D). For passenger strand cleavage, hAgo2 and the different dsRBPs were pre-incubated and the reaction was started by adding double stranded siRNA (as2b/s2b) comprising a radioactively labelled passenger strand. For short target RNA cleavage hAgo2, single-stranded siRNA (as2b) and the different dsRBPs were pre-incubated and the reaction was started by adding the guide-matching 21 nt radio-labelled target RNA s2b. In case of passenger strand cleavage, we observed an increase of the cleavage amplitude from about 20% to about ≥90% in the presence of the respective dsRBP. Interestingly, contrary to what we observed for the 140 nt target RNA, cleavage of the short 21 nt target is similarly positively affected already in case of a single-stranded siRNA. Comparing all the cleavage reactions, we found that dsRBPs drive the amplitude of cleavage to a distinct level of 70–100% regardless of the level of cleavage in the absence of dsRBPs (Table 3).

**Table 3:** Rate constants and amplitudes of hAgo2-mediated RNA cleavage in presence of dsRNA-binding proteins.

	Passenger cleavage	ss-siRNA	ds-siRNA	ss-siRNA	
		21-nt target	140-nt target	140-nt target	
***k*:**	0.0001 (±5x10^-5^) s^-1^	0.0001 (±6x10^-5^) s^-1^	4 x 10^−5^ (±2x10^-5^) s^-1^	0.0005 (±9x10^-5^) s^-1^	**w/o dsRBP**
**A:**	19.8 (±6.7)%	18.6 (±9) %	17.8 (±6.4) %	85.7 (±4.4) %	
***k*:**	0.0002 (±9x10^-6^) s^-1^	0.0007 (±2x10^-4^) s^-1^	0.0003 (±1x10^-5^) s^-1^	0.0006 (±2x10^-4^) s^-1^	**w/h TRBP**
**A:**	87.7 (±2.0) %	73.3 (±5.8) %	67.0 (±0.9) %	68.4 (±9.9) %	
***k*:**	0.002 (±2x10^-4^) s^-1^	0.003 (±2x10^-4^) s^-1^	0.0003 (±6x10^-5^) s^-1^	0.003 (±4x10^-4^) s^-1^	**w/h TRBP-D12**
**A:**	88.9 (±1.1) %	75.5 (±1.6) %	82.2 (±6.6) %	79.1 (±2.6) %	
***k*:**	0.0002 (±3x10^-5^) s^-1^	0.0006 (±2x10^-4^) s^-1^	0.0001 (±7x10^-6^) s^-1^	0.0008 (±1x10^-4^) s^-1^	**w/h PACT**
**A:**	98.4 (±4.2) %	90.3 (±4.9) %	95.2 (±5) %	89.4 (±2.3) %	

*k*: rate constant, A: amplitude. Rate constants and amplitudes are averaged from at least three independent measurements. Standard deviations in brackets. For corresponding plots see Fig 3.

**Fig 4:**
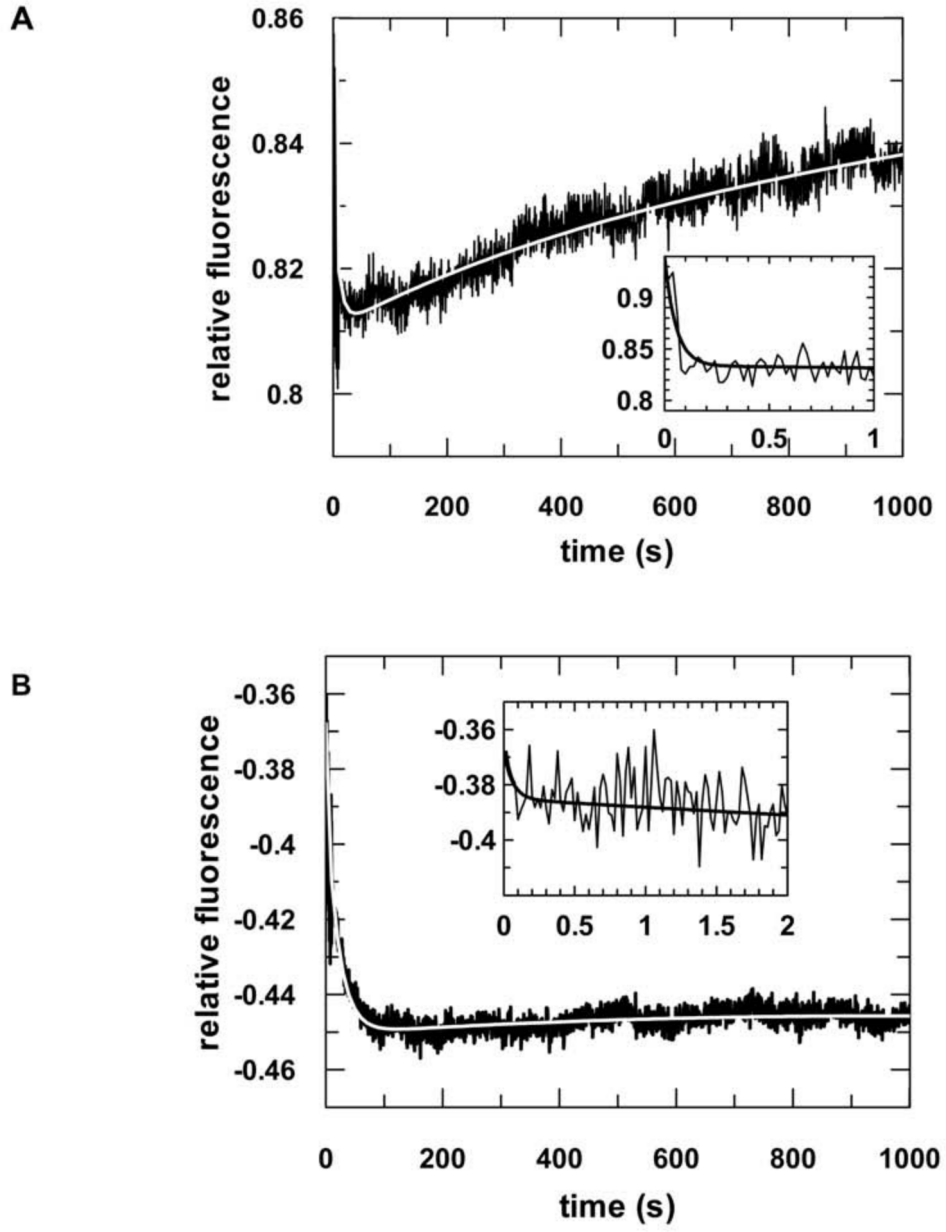
Dissociation of ternary complexes in presence of hTRBP-D12 or hTRBP. Representative stopped-flow graphs are shown. Ternary complexes composed of 500 nM hAgo2, 20 nM guide RNA (as2b-FAM) and 40 nM target RNA (s2b) were preassembled and subsequently rapidly mixed with 2 μM unlabeled guide RNA and 500 nM hTRBP-D12 (A) or hTRBP (B). In both cases data could be best fitted using a triple exponential equation, yielding the following rate constants: (A) *k*_-1_: 18.4 (± 1.2) s^-1^, *k*_-2_: 0.1 (± 0.007) s^-1^, *k*_-3_: 0.002 (± 0.0005) s^-1^ and (B) *k*_-1_: 15.9 (± 4.6) s^-1^, *k*_-2_: 0.05 (± 0.001) s^-1^, *k*_-3_: 0.0005 (± 0.0002) s^-1^. Rate constants are summarized in Table 4.

Regarding hTRBP-D12, not only did the cleavage amplitude increase in both situations (i.e. passenger strand and short target RNA), but also the cleavage rate went up by a factor of 10. Since product release represents the overall rate-limiting step of target cleavage under multiple turnover conditions [27–30], we reasoned hTRBP-D12 might enhance target dissociation from ternary hAgo2/guide/target complexes. To test this assumption, we analyzed dissociation of preassembled ternary hAgo2/guide/target complexes in presence of hTRBP-D12 or hTRBP (Fig 4). Previous studies revealed the dissociation of target RNA from ternary complexes as a three-step process involving guide/target strand separation in the 3’- as well as 5’-region (i.e. seed region) of the guide [27]. In presence of hTRBP-D12, these two steps of target release are accelerated by 10- and 45-fold, respectively (Table 4). Even though no effect of hTRBP on the cleavage rate could be detected, pre-steady state binding measurements clearly indicate that hTRBP also influences target dissociation from ternary complexes. Here, strand separation in the seed region of the guide strand (*k*_-2_) is accelerated about 30-fold (Table 4). These different effects of hTRBP with or without the third domain suggest the dsRBD3 affects the exact positioning of the protein within the hAgo2/nucleic acid complex. Moreover, hTRBP evidently modulates dissociation of cleavage products.

**Table 4:** Dissociation of ternary hAgo2/guide/target complexes in presence of hTRBP and hTRBP-D12.

	*k*_-1_ (s^-1^)	*k*_-2_ (s^-1^)	*k*_-3_ (s^-1^)
hAgo2 w/o dsRBP*	2 (± 0.25)	0.002 (± 0.0002)	0.0002 (± 6x10^-5^)
**hAgo2 w/ hTRBP**	16 (± 0.1)	0.06 (± 0.01)	0.0004 (± 0.0002)
**hAgo2 w/ hTRBP-D12**	21.5 (± 4.3)	0.09 (± 0.01)	0.002 (± 0.0001)

* data taken from Deerberg et al. [27]. Rate constants are averaged from up to three independent measurements. Standard deviations in brackets. For corresponding plots see Fig 4.

### hTRBP and hPACT affect siRNA binding to hAgo2

Recently, we have shown that formation of binary hAgo2-siRNA complexes is at least a three-step process. The first step represents collision complex formation of the two binding partners, whereas the second and third step correspond to siRNA 5’-anchoring in the MID-domain and 3'-binding to the PAZ-domain, respectively [27]. To investigate if the two dsRNA-binding proteins have an effect on binary hAgo2/siRNA complex assembly, we performed pre-steady state binding experiments in the presence of hTRBP, hPACT or hTRBP-D12 using a stopped-flow apparatus.

**Fig 5:**
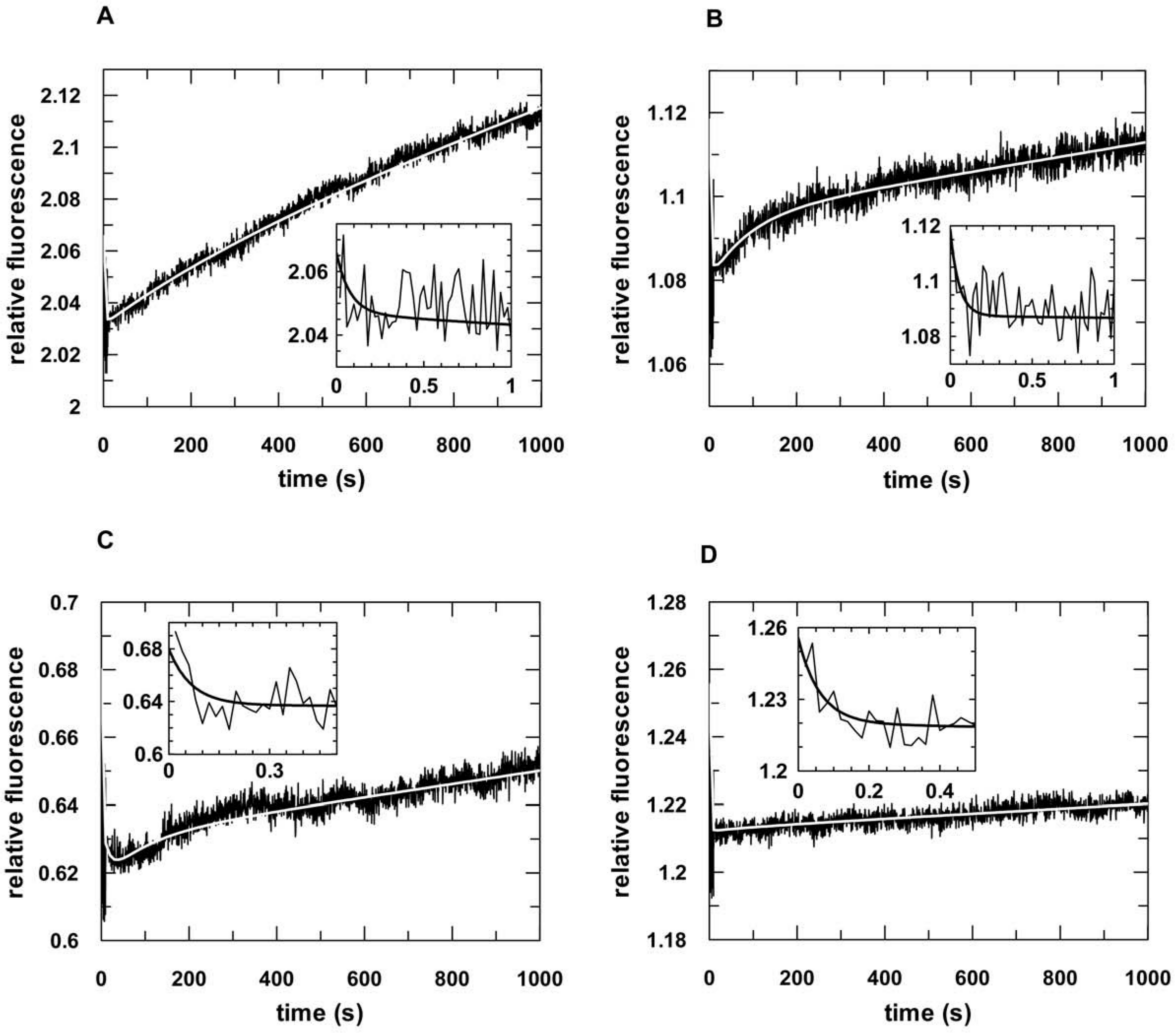
Pre-steady state kinetics of the effect of hTRBP and hPACT on complex formation between hAgo2 and double-stranded siRNA. Representative stopped-flow graphs are shown. The inserts show the reaction on a shorter time scale. 20 nM of fluorescently labeled double-stranded siRNA (as2b-FAM/s2B) were pre-incubated with 600 nM hTRBP (A, C) or hPACT (B, D). The binary complexes were subsequently rapidly mixed with either hAgo2 (400 nM in case of A and 600 nM in case of B) or 600 nM hAgo2-PAZ9 (C, D). Data were fitted to an exponential equation. For hAgo2 & hTRBP: *k*_1_: 28.8 (± 10.8) s^-1^, *k*_2_: 0.33 (±0.03) s^-1^ and *k*_3_: 0.002 (± 0.0001) s^-1^ and for hAgo2 & hPACT: *k*_1_: 18.6 (± 3.5) s^-1^, *k*_2_: 0.13 (± 0.02) s^-1^ and *k*_3_: 0.01 (± 0.001) s^-1^ were determined. With the mutant hAgo2-PAZ9 the following rate constant were calculated: *k*_1_: 14.9 (± 2.7) s^-1^, *k*_2_: 0.07 (±0.01) s^-1^ and *k*_3_: 0.01 (± 0.0009) s^-1^ in the presence of hTRBP and *k*_1_: 16.2 (± 0.6) s^-1^, *k*_2_: 0.38 (±0.01) s^-1^ and *k*_3_: 0.007 (± 0.002) s^-1^ in the presence of hPACT.

As substrate, we used a double-stranded siRNA with a FAM-label at position 14 of the guide strand (as2b-FAM/s2b) as described before [27]. This siRNA was preincubated with one of the dsRNA-binding proteins and subsequently mixed with hAgo2. Equivalent to binary complex formation in absence of dsRBPs, we observed a three-step binding process (Fig 5 and Table 5). The resulting first and second forward rate constants more or less match those obtained in absence of dsRBPs. Interestingly, while in the presence of hPACT, the third forward rate is reduced by about 4-fold, it is further slowed down in presence of hTRBP by a factor of 8 and even more with hTRBP-D12, namely by a factor of 15. This implies hTRBP might directly interact with the PAZ domain or in a region close by where it could influence PAZ binding of the siRNA. However, as hPACT likewise enhances the hAgo2-mediated cleavage, this interpretation cannot be the sole reason for the observed dsRBP-induced effect.

**Table 5:** Association of hAgo2/siRNA complexes in presence of dsRBPs.

	*k*_1_ (M^-1^ s^-1^)	*k*_2_ (s^-1^)	*k*_3_ (s^-1^)
hAgo2 w/o dsRBP*	1.0 (± 0.13) x 10^8^	0.48 (± 0.23)	0.03 (± 0.02)
**hAgo2 w/hTRBP**	0.2 (± 0.009) x 10^8^	0.2 (± 0.1)	0.004 (± 0.001)
**hAgo2 w/hTRBP-D12**	0.3 (± 0.04) x 10^8^	0.4 (± 0.19)	0.002 (± 0.0006)
**hAgo2 w/hPACT**	0.5 (± 0.09) x 10^8^	0.12 (± 0.02)	0.008 (± 0.003)
**hAgo2-PAZ9**	0.2 (± 0.03) x 10^8^	0.43 (± 0.08)	-
**hAgo2-PAZ9 w/hTRBP**	n. d.	0.1 (± 0.05)	0.01 (± 0.0008)
**hAgo2-PAZ9 w/hPACT**	n. d.	0.35 (± 0.06)	0.005 (± 0.002)

* data taken from Deerberg et al. [27]. Rate constants are averaged from up to five independent measurements. Standard deviations in brackets. For *k*_1_ standard errors are given.

**Fig 6:**
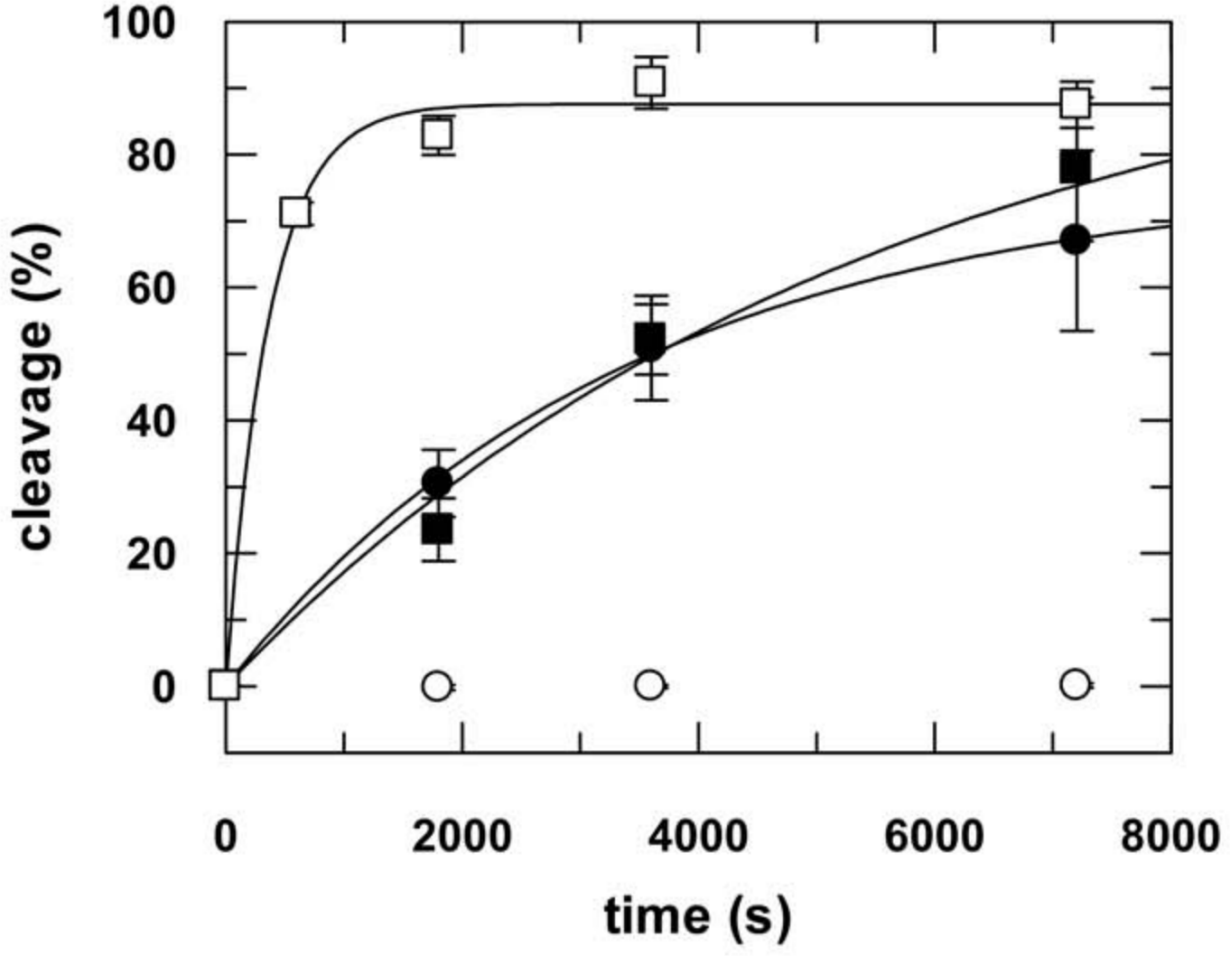
hAgo2-PAZ9-mediated RNA cleavage. Cleavage reaction was started by adding double-stranded 5’-^32^P-passenger labeled siRNA (as2b/s2b) to a reaction mix containing hAgo2-PAZ9 alone (open circles) or hAgo2-PAZ9 in the presence of either hTRBP (closed circles), hPACT (closed squares) or hTRBP-D12 (open squares). Samples were collected at different time points, analyzed by 20% denaturing PAGE and detected by autoradiography (Figure J in S1 Supporting Information File). Without dsRBPs no cleavage activity is recognizable, whereas in case of hTRBP, hPACT and hTRBP-D12 cleavage amplitudes of 76.6 (± 2.2) %, 103.3 (± 9.2) % and 87.6 (± 1.3) % could be observed. The corresponding rate constants are 0.0003 (± 0.00002) s^-1^, 0.0002 (± 0.00003) s^-1^ and 0.003 (± 0.0003) s^-1^, respectively. Data points are averaged from three independent measurements. Error bars represent standard deviation.

To further elucidate the effect of dsRBPs on hAgo2/siRNA interaction, we made use of the hAgo2-PAZ9 mutant. This mutant contains nine point mutations in the PAZ domain and shows impaired binding of siRNA and virtually no target RNA cleavage activity [31]. First, we tested passenger strand cleavage activity of hAgo2-PAZ9 in absence and presence of dsRBPs (Fig 6). Astoundingly, while as expected without dsRBPs no cleavage could be detected, the addition of either hTRBP, hPACT or hTRBP-D12 rescued passenger cleavage by boosting the amplitude of product formation from 0 to 80–100%. These amplitudes are comparable to those obtained with hAgo2 in presence of any of the three dsRBPs. Moreover, the rate constant of cleavage is indistinguishable from wt enzyme. This clearly indicates that the binding of siRNAs to hAgo2-PAZ9 is restored by dsRBPs. Furthermore, this observation corroborates the thesis that dsRBPs drive the cleavage amplitude to a specific level rather than accelerating the reaction by a fixed multiplier.

To confirm the assumption dsRBPs not only assist in binding of siRNAs to hAgo2 but are also able to rescue binding of siRNAs to binding-deficient hAgo2, we performed pre-steady state siRNA binding experiments with hAgo2-PAZ9. In the absence of dsRBPs, we exclusively observed two phases, namely collision complex formation (*k*_1_) and siRNA 5’-anchoring in the MID-domain (*k*_2_), while 3'-binding to the PAZ-domain (*k*_3_) could not be detected (Table 5). However, addition of any of the dsRBPs fully restored the three-step mechanism of siRNA-binding (Fig 5). Overall, these results clearly point out that dsRBPs play an important role for the positioning and binding of the siRNA and therefore lead to efficient hAgo2-mediated target RNA cleavage. Since hAgo2-PAZ9 is not able to bind the 3’-end of siRNA on its own, we propose that the dsRBPs bind to hAgo2 and siRNA in a clamp like fashion to fix the duplex in the proper position for cleavage to occur.

### hTRBP and hPACT bind to the Ago2-siRNA complex in different ways to enhance cleavage

**Fig 7:**
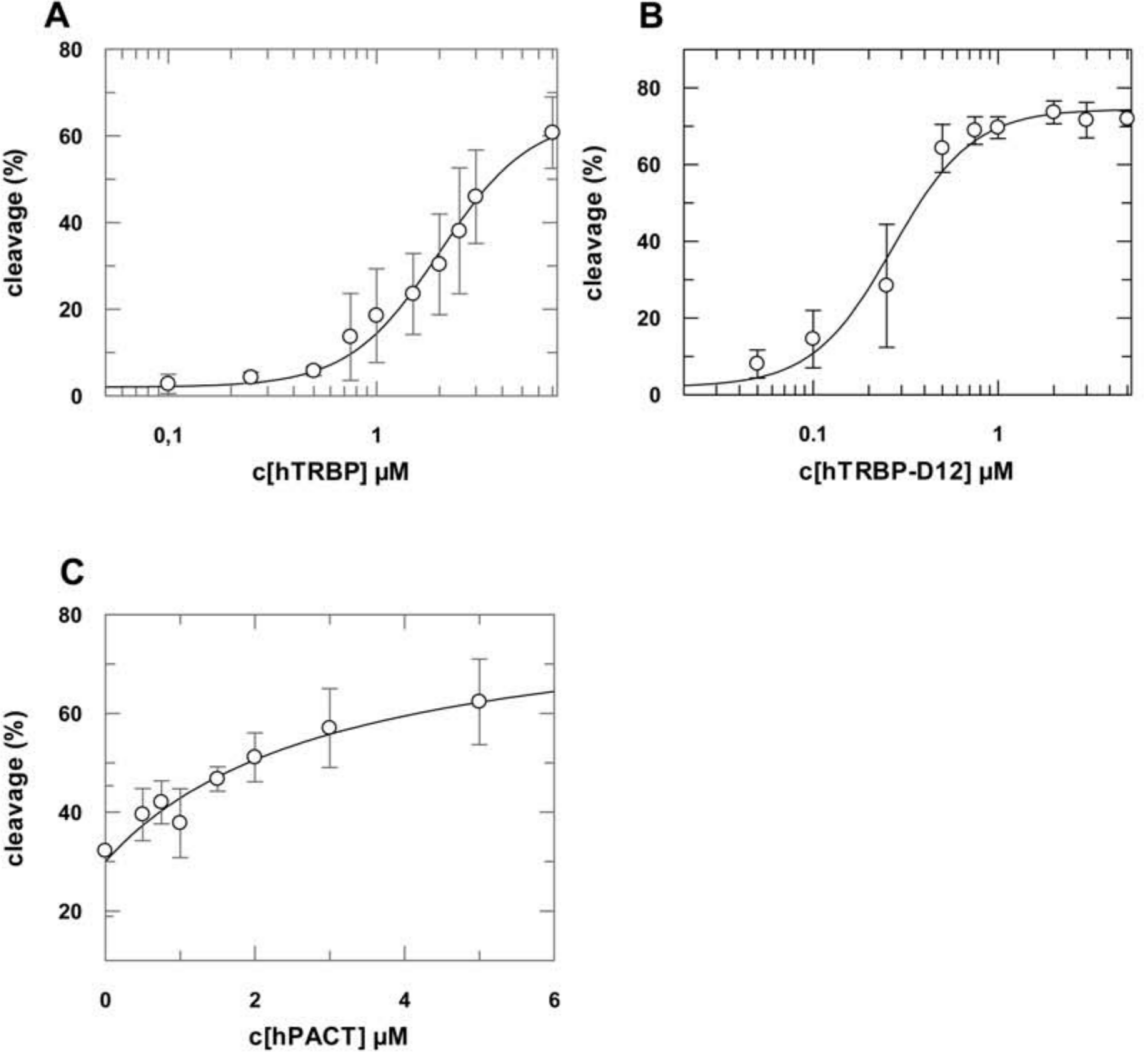
hAgo2-catalyzed RNA cleavage as a function of the concentration of dsRNA-binding proteins. 3 μM of hAgo2 were pre-incubated with increasing concentrations of hTRBP (A), hTRBP-D12 (B) or hPACT (C), respectively. Cleavage reactions were started by adding 5’-^32^P-passenger labeled siRNA (as2b/s2b) at a final concentration of 40 nM. Samples were incubated at 37°C for 2 h. Experimental data were fitted to a Hill equation yielding *K*_*ds*_ of 2.0 (± 0.2) μM (A) and 0.3 (± 0.03) μM (B) with a Hill coefficient of 2. For hPACT a *K*_d_ of 3.0 (± 1.0) μM (C) with Hill coefficient of 1 was determined. Data shown are averaged from at least three individual measurements. Error bars represent standard deviations. Corresponding gels are shown in Figure K in S1 Supporting Information File.

Our data, further corroborated by pulldown assays (Figure I in S1 Supporting Information File), indicate a direct physical interaction between hAgo2 and dsRBPs. To investigate this issue in more detail, we performed passenger cleavage experiments with gradually increasing hTRBP concentrations from 0 μM to 7 μM. Here, the boost of cleavage activity reaches 50% of its maximum level at a hTRBP concentration of 2.5 μM (Fig 7A). The corresponding experimental data could be best fitted to Hill`s equation yielding a Hill coefficient of 2 and a *K*_d_ of 2 μM for the interaction between hTRBP and the hAgo2/siRNA complex. This result indicates there might be more than one binding site for hTRBP within the hAgo2/siRNA complex which enables cooperative binding of hTRBP.

Likewise, the deletion mutant hTRBP-D12 enhances the hAgo2-mediated RNA cleavage in a concentration dependent manner (Fig 7B). In comparison to hTRBP, it reaches the maximum level of cleavage activity already at a concentration of 0.75 μM. Fitting of the data to Hill’s equation yielded a Hill coefficient of 2. This is a strong hint that although positioning of wt hTRBP and hTRBP-D12 might be different, the modes of binding are similar. Since the stimulation effect of the C-terminal deletion mutant is more pronounced compared to the full-length protein, we conclude that the C-terminal domain might have a regulatory effect. The equilibrium binding constant for hTRBP-D12 and hAgo2/siRNA complexes amounts to 0.3 μM (Fig 7B).

In contrast to hTRBP and its deletion mutant, hPACT does not bind to the hAgo2/siRNA complex in a cooperative manner (Fig 7C). The examination of concentration dependency of hPACT on the hAgo2-mediated passenger cleavage was conducted as described above for hTRBP. Data could be best fitted to Hill’s equation with a Hill coefficient of 1 which is the signature of a non-cooperative binding event. The *K*_*d*_ for the binding between hPACT and the hAgo2/siRNA complex is 3 μM.

## Discussion

The dsRNA-binding proteins TRBP and PACT have been shown to contribute to RNAi in many different ways [7,15,16,24,25,32–38]. Our goal was to analyze potential direct effects of these dsRNA-binding proteins on hAgo2-mediated siRNA binding and cleavage of passenger strands or corresponding target RNAs. A recently presented minimal mechanistic model of hAgo2-mediated RNA slicing [27] enabled us to directly assign the effect of hTRBP and hPACT to different steps of siRNA binding and cleavage of target RNAs. We found that hTRBP as well as hPACT are key components that modulate the efficiency of hAgo2-mediated target RNA cleavage.

Equilibrium analyses of the interaction between siRNA and hTRBP or hPACT yielded similar *K*_*d*_ values in the low nanomolar range for both proteins (Table 2). On the one hand, this is in contrast to findings of Takahashi et al. [39] who reported a *K*_*d*_ of hPACT for siRNA of 200–400 nM. On the other hand, the equilibrium dissociation constant for hTRBP and siRNA fits reasonably well with previously determined *K*_*d*_ values [17,39]. While Parker et al. described a one-site binding mode for the hTRBP/siRNA interaction, Yamashita et al. as well as Takahashi et al. reported a two-site binding mode and hence determined a second *K*_*d*_ value that is significantly higher than the first one [17,39,40]. The reason we also observe only a single binding mode could be explained by our experimental setup; i.e. fluorophore attached to the 5'-end of the guide strand. It has been described previously, hTRBP preferentially binds, at least initially, to the ends of siRNAs [36] and moreover binding of individual proteins to a single nucleic acid occurs non-cooperatively [40]. Accordingly, in our experimental setup we might not expect to observe additional changes in fluorescence upon protein/nucleic acid interactions even if several binding events per siRNA molecule take place in parallel or consecutively, representing either binding of the second dsRNA-binding domain of the same molecule or binding of an additional unrelated molecule. Unfortunately, attempts to change the position of the fluorophore on the siRNA to potentially characterize such binding events did not prove successful.

Transient kinetic analyses of the assembly of hTRBP or hPACT with siRNA revealed a binding process comprising of at least two steps with individual rate constants being almost indistinguishable for the two dsRNA-binding proteins (Table 2). The first step observed represents diffusion-controlled formation of a collision complex between siRNA and protein, while the second step corresponds to a conformational rearrangement leading to tight binding. However, at present we cannot precisely resolve the exact nature of this conformational rearrangement. Most likely it represents the interaction of at least one of the dsRBDs with the siRNA or a rate-limiting step given by conformational changes within the linker regions. Along the lines given above, we would not expect to experimentally resolve consecutive binding of the second dsRBD to the same siRNA molecule. Alternatively, it is also conceivable that both dsRBDs bind simultaneously to the siRNA as suggested by others [17,39].

To begin with, binding experiments (equilibrium as well as transient) were performed with recombinant proteins purified under denaturing conditions followed by refolding. The numbers obtained from transient binding studies are almost identical for the two proteins (Table 2). However, these data differ substantially from those measured for proteins strictly purified under native conditions while the measured equilibrium constants are remarkably the same (Table 2). The discrepancy in the second order rate constant (*k*_+1_) resembling initial complex formation by about 10-fold is most likely due to a slight aggregation of proteins purified under native conditions causing reduced diffusion. Attempts to fully remove such aggregates proved technically not feasible since there is a dynamic equilibrium between monomeric and oligomeric structures. Interestingly, the second transition which as outlined above, most likely resembles conformational rearrangements within the dsRBDs is 90–170-fold slower in case of hTRBP^nat^ and hPACT^nat^. Moreover, dissociation of binary protein/nucleic acid complexes is reduced by 5–20-fold in case of hTRBP^nat^. For PACT^nat^ we were unable to reliably measure nucleic acid dissociation most likely due to protein/nucleic acid aggregation. Taken together, these observations imply, albeit the overall binding affinity for dsRNA does not seem to be affected, the two proteins behave entirely different depending on the purification protocol applied. As outlined in the experimental section, additional experiments finally revealed protein purified under denaturing conditions to be functionally crippled. Accordingly, all further experiments were performed with protein purified under native conditions. This is an excellent example that a detailed transient binding study is capable of disclosing substantial differences within different protein preparations which otherwise might have not been detected.

Both dsRBPs did not show major differences in their siRNA binding behavior and their effects on hAgo2-mediated RNA cleavage are in parts comparable. hTRBP as well as hPACT lead to a strong increase of cleavage product formation in case of siRNA-mediated target and passenger RNA cleavage. Interestingly, such a stimulating effect of hTRBP and hPACT on the cleavage of long target RNA could not be found with hAgo2 programmed with single-stranded guide RNA. On the contrary, we observed a decrease of product formation when adding hTRBP to cleavage reactions with single-stranded siRNA and long target RNA. Therefore, we conclude that siRNA binding of hTRBP and hPACT is a precondition for their cleavage enhancing abilities. Then again, both hTRBP and hPACT cause an increase of cleavage product formation when added to cleavage reactions with single-stranded siRNA and short target RNA. This leads to the proposition that a combination of single-stranded siRNA and short target RNA, contrary to the situation in case of a long target RNA, resembles binding of a double-stranded siRNA. Moreover, this implies considerable differences between these two types of complexes; i.e. single-stranded siRNA bound to a short or long target RNA.

We found that hTRBP-D12 stimulates the cleavage activity even more than hTRBP does and also increases the cleavage rate by a factor of 10. Analyses of ternary complex dissociation in presence of hTRBP or hTRBP-D12 revealed a role of both proteins in target release. While hTRBP-D12 is accelerating the target dissociation in the seed (*k*_-2_) as well as the 3’-region (*k*_-3_) of the guide, full-length hTRBP leads to an acceleration of the target RNA dissociation in the seed region (*k*_-2_) of the guide. Considering the seed region of the guide being pre-arranged for base pairing within a binary complex [41–43], dissociated product strands might quickly rebind to a hAgo2/guide RNA complex. Thus, a possible biological role of hTRBP could be clearance of the seed region from cleavage products to ensure effective binding of the next incoming target. Since hTRBP-D12 is accelerating dissociation in both the seed as well as in the 3’-region, we conclude that the dsRBD3 is involved in the exact positioning of hTRBP within the hAgo2/nucleic acid complex. These results further indicate that the dsRBD3 has a rather regulative function, whereas the actual stimulating effect of the two proteins in turn is mediated via the dsRBDs 1 & 2.

This leads to the question of how hTRBP and hPACT enhance cleavage product formation by hAgo2. We found that neither of the dsRBPs shows a considerable effect on the assembly of wt-hAgo2 and siRNA. However, if we perform such experiments with the RNA binding impaired hAgo2-PAZ9 mutant [31] instead, we find that both hTRBP and hPACT fully rescue siRNA binding and as a result cleavage activity. Since this mutant does not bind the siRNA 3’-end on its own, we conclude that the dsRBP need to stay bound to hAgo2 during the actual cleavage reaction to secure optimal siRNA positioning. As a result, it appears inevitable dsRBP and siRNA duplex are bound simultaneously by hAgo2 during the entire enzymatic cycle. For this activity, apparently the first two dsRBDs of hTRBP are sufficient. This confirms the conception these two dsRBDs are indispensable for the observed stimulative effect, whereas the non-RNA binding dsRBD3 [17] appears to possess a regulatory function. Although hAgo2 is able to bind siRNAs as well as miRNAs [27,44] and subsequently mediate siRNA-dependent target RNA cleavage on its own [27], hTRBP and hPACT seem to be important regulators that promote target cleavage efficiency. Intriguingly, although hPACT and hTRBP confer a similar effect on the hAgo2-mediated cleavage, their mode of action is different. By investigating dsRBP concentration dependency of hAgo2-mediated passenger cleavage, we found that hTRBP is binding to hAgo2 in a cooperative manner, whereas binding of hPACT was found to be non-cooperative. It was shown before that binding of hTRBP to dsRNA is non-cooperative [40] and the stoichiometry found in the RLC between hDicer, hAgo2 and hTRBP is 1:1:1 [10]. This might indicate the following: (i), after dissociation of hDicer from the RLC, additional sites on hAgo2 become available for hTRBP to bind, which along with our other findings leads to the conclusion that final positioning of siRNA within hAgo2 only takes place after dissociation of hDicer. (ii), it provides additional evidence that for proper positioning of siRNA within the binary hAgo2 complex, a direct protein/protein interaction between the dsRBP and hAgo2 is crucial since binding to siRNA in absence of hAgo2 is significantly tighter (Fig 1) and moreover non-cooperative [40]. The concept of a direct protein/protein interaction between hAgo2 and hTRBP was recently confirmed by Chen et al. who collected evidence TRBP is required for regulating miRNA/siRNA efficiency in vivo by interacting with hAgo2 [45].

Overall, our results show that hTRBP and hPACT are involved in regulation of siRNA-mediated RNAi and are critical for efficient target RNA cleavage by directly interacting with hAgo2.

## Materials and Methods

### RNA

The short RNAs as2b, s2b, aslam and slam were ordered from IBA or Biomers. ICAM-1 *in vitro* transcript (ICAM-1-IVT) was transcribed from plasmid pG-si2b (+) using T7 RiboMAX™ Express Large Scale RNA Production System (Promega) and afterwards purified using phenol-chloroform extraction and ethanol precipitation. Aslam-FAM and slam, as well as as2b and s2b or as2b-FAM and s2b were annealed in order to obtain siRNA-like duplexes. All nucleic acid sequences used in the present study are listed in Table 1.

As2b as the guide strand was phosphorylated at the 5’-end with unlabeled ATP (Fermentas), while ICAM-1-IVT and s2b were 5’-phosphorylated with [γ-^32^P] ATP (Hartmann, PerkinElmer) where appropriate. The modified RNAs were purified by Sephadex-G50 columns (GE Healthcare), phenol-chloroform extraction and ethanol precipitation. Complementary RNAs were annealed using equimolar amounts of both strands in siRNA annealing buffer (15 mM HEPES pH 7.4, 50 mM KCH_3_COOH, 1 mM MgCH_3_COOH). The two strands were incubated for 3 min at 95°C and slowly cooled down to room temperature. The integrity of the hybridized siRNA was tested using native PAGE-analysis visualized by using radioactive- or FAM-labelled RNAs or by staining with Stains-All (Sigma-Aldrich).

### Preparation of GST-hAgo2, GST-hAgo2-PAZ9, His-hTRBP, His-hPACT and His-hTRBP-D12 for biochemical analysis

GST-hAgo2 as well as GST-hAgo2-PAZ9 were expressed and purified as previously described [27]. The hTRBP deletion mutant hTRBP-D12 was created with the overlap PCR method [46]; residues 297–349 were deleted. Full-length hTRBP as well as the deletion mutant hTRBP-D12 and hPACT were cloned into expression vector pET41b (+) (Merck). This vector was transformed into *E*. *coli* strain BL21 (DE3). Purification of hTRBP and hPACT was performed under native as well as denaturing conditions (Figure A in S1 Supporting Information File). hTRBP-D12 was exclusively purified under native conditions. Subsequent description applies to all three proteins. *E*. *coli* cells were induced with 1 mM IPTG at OD_600_ 1.0 and harvested after either 1h (native protocol) or 4h (denaturing protocol) incubation at 37°C at 200 rpm. Cell lysis was performed in 20 mM Tris, pH 7.5, 50 mM NaCl, 1 mM PMSF, 5 mM ß-mercaptoethanol and 5 mM imidazole (native) or 50 mM NaH_2_PO_4_ pH 7.6, 300 mM NaCl, 1 mM Tris(2-carboxyethyl)phosphine and 7 M urea (denaturing). At first the cells were sonicated in 5 iterative rounds for 20 seconds at 70% power followed by 30 seconds break (Sonopuls UW/GM 70, Bandelin). Afterwards, in case of the native protocol the cells were incubated with Lysozyme (100 μg/ml) for 15 min at 4°C. Subsequently, the sonication protocol was repeated 10 times. His-tagged proteins were purified via affinity chromatography using Talon *Superflow* (Takara Clontech). After washing the column with buffer A (native: 20 mM Tris, pH 7.5, 50 mM NaCl, 1 mM PMSF, 5 mM imidazole) or A’ (denaturing: 50 mM NaH_2_PO_4_ pH 7.6, 300 mM NaCl, 1 mM Tris(2-carboxyethyl)phosphine and 7 M urea), the protein was eluted with buffer B (native: 20 mM Tris, pH 7.5, 200 mM imidazole, 50 mM NaCl) or B’ (denaturing: 50 mM NaH_2_PO_4_ pH 7.6, 300 mM NaCl, 1 mM Tris(2-carboxyethyl)phosphine, 500 mM imidazole and 7 M urea). The purified native protein was dialyzed against buffer C (20 mM Tris, pH 7.5, 50 mM NaCl, 10% glycerol, 1 mM EDTA). In case of the denatured proteins, a series of in total seven dialysis steps of two hours each were performed gradually using decreasing urea and NaCl concentrations (step1: 4 M urea and 200 mM NaCl, step2: 4 M urea and 100 mM NaCl, step3: 3 M urea and 100 mM NaCl, step4: 2 M urea and 100 mM NaCl, step5: 1 M urea and 100 mM NaCl, step6: 0.5 M urea and 100 mM NaCl, step7: 100 mM NaCl). Finally, proteins were stored in buffer C’ (20 mM Tris pH 8.0, 100 mM NaCl, 10% glycerol and 1 mM DTT).

### RNA cleavage assay

RNA cleavage assays were performed using 3.0 μM GST-hAgo2, 100 nM siRNA and 2.5 nM radiolabeled target RNA or 3.0 μM GST-hAgo2 and 30 nM siRNA with a radiolabeled passenger strand. ICAM-1-IVT (140 nt) or s2b (21 nt) served as target RNA, while si2b (hybrid of as2b and s2b) was used as siRNA. Despite the experimental setup described might suggest otherwise we do indeed observe multiple turnover under such conditions [27]. The components were incubated at 37°C in 1x cleavage buffer (10 mM Tris, pH 7.5, 100 mM KCl, 2 mM MgCl_2_). The cleavage reaction was started by adding target RNA or siRNA when passenger cleavage was analyzed. Samples were taken at different time points. The reaction was stopped using formamide buffer (95% formamide (v/v), 0.025% (w/v) SDS, 0.025% (w/v) bromphenolblue, 0.025% (w/v) xylencyanol, 0.5 mM EDTA). The samples were separated on sequencing gels under denaturing conditions (7 M urea). Subsequently, the gel was dried in a vacuum dryer and detected using PhosphorImager Typhoon^TM^ 8600 (Amersham Pharmacia). The amount of cleavage product and full-length target RNA or passenger RNA was determined using Image Quant 5.2 (Amersham Pharmacia). Data were fitted using a single exponential equation (Grafit 5, Erithacus Software).

### Fluorescence-based steady state measurements

Steady state measurements were performed with the fluorescence spectrometer FluoroMax^R^-3 (Horiba Jobin Yvon). To analyze the binding reaction of hTRBP or hPACT to siRNA, 50 nM of FAM-labelled siRNA were mixed with increasing concentrations of the respective protein in TRBP assay buffer (20 mM Tris pH 7.5, 50 mM NaCl, 1 mM EDTA) at 25°C. The fluorophore was excited at 492 nm, while the emitted light was detected at 516 nm. To determine the equilibrium dissociation constant of the protein/RNA interactions, data were evaluated using GraFit 5 (Erithacus Software). The data were fit by a quadratic equation of the form: Fluorescence = F_max_-((c[siRNA]+L+*K*_d_)-sqrt((sqr(c[siRNA]+L+*K*_d_))-4*c[siRNA]*L))*(F_max_ F_min_)/((2*c[siRNA])). F_max_ and F_min_ represent the maximal and the minimal fluorescence intensity, L is the concentration of dsRNA-binding protein and *K*_d_ is the equilibrium dissociation constant.

### Fluorescence-based pre-steady state measurements

Association and dissociation rate constants of protein/RNA complexes were determined with the stopped flow system SX20 (Applied Photophysics) at 25°C. The FAM-Label of the siRNA was excited at 490 nm and the emitted light was detected with a cut-off filter at wavelengths above 530 nm. The binding reaction was triggered by rapid mixing (~ 2 ms) of dsRNA-binding protein and 5’-FAM-labeled siRNA in assay buffer (20 mM Tris pH 7.5, 50 mM NaCl, 1 mM EDTA). When binary hAgo2/siRNA complex formation was analyzed in the presence of a dsRNA-binding protein, the corresponding dsRNA-binding protein was preassembled with the siRNA (here FAM label at position 14) before mixing with Ago2 in Ago assay buffer (10 mM Tris pH 7.5, 100 mM KCl, 0.5 mM MgCl_2_). For the dissociation reaction, protein and FAM-labeled siRNA were pre-assembled and mixed with a 7-fold excess of unlabeled siRNA. The experimental data were evaluated with GraFit 5 (Erithacus Software) in order to obtain rate constants for the different steps of the binding reaction. An exponential equation "Fluorescence = ∑A_n_*exp(-*k*_n_*t)" was used to fit the data. In this equation, *k*_n_ represents the rate constant of the observed phase, A_n_ stands for the amplitude of the phase and t represents the time.

## Supporting Information

S1 Supporting Information File File includes Figures A—K. Figure A. SDS-PAGE analysis of purified recombinant proteins, Figure B. Equilibrium titrations of hTRBPdenat and hPACTdenat with double-stranded siRNA, Figure C. Pre-steady state kinetic analyses of the association of hTRBPdenat or hPACTdenat and double-stranded siRNA, Figure D. Concentration dependencies of the first phase of double-strand siRNA binding (observed pseudo-first-order rate constant) with hTRBPdenat and hPACTdenat are shown, Figure E. Concentration dependencies of the first phase of double-strand siRNA binding (observed pseudo-first-order rate constant) with hTRBPnat and hPACTnat are shown, Figure F. Kinetics of the dissociation of hTRBPnat/ds-siRNA-complexes, Figure G. Kinetics of the dissociation of hTRBPdenat/- or hPACTdenat/ds-siRNA-complexes, Figure H. hAgo2-mediated cleavage of target RNA in presence or absence of dsRNA-binding proteins, Figure I. Detection of protein-protein interactions between hAgo2 and hPACT or hTRBP, Figure J. hAgo2-PAZ9-mediated RNA cleavage and Figure K. hAgo2-catalyzed RNA cleavage as a function of the concentration of dsRNA-binding proteins.
(PDF)
