# Supplementary material for: Recombinant hTRBP and hPACT Modulate hAgo2-Catalyzed siRNA-Mediated Target RNA Cleavage In Vitro
Source: PLoS One. 2016 Jan 19;11(1):e0146814. doi: 10.1371/journal.pone.0146814 (PMC4718636; doi:10.1371/journal.pone.0146814)
Supplement: S1 Supporting Information File — Figure A. SDS-PAGE analysis of purified recombinant proteins, Figure B. Equilibrium titrations of hTRBPdenat and hPACTdenat with double-stranded siRNA, Figure C. Pre-steady state kinetic analyses of the association of hTRBPdenat or hPACTdenat and double-stranded siRNA, Figure D. Concentration dependencies of the first phase of double-strand siRNA binding (observed pseudo-first-order rate constant) with hTRBPdenat and hPACTdenat are shown, Figure E. Concentration dependencies of the first phase of double-strand siRNA binding (observed pseudo-first-order rate constant) with hTRBPnat and hPACTnat are shown, Figure F. Kinetics of the dissociation of hTRBPnat/ds-siRNA-complexes, Figure G. Kinetics of the dissociation of hTRBPdenat/- or hPACTdenat/ds-siRNA-complexes, Figure H. hAgo2-mediated cleavage of target RNA in presence or absence of dsRNA-binding proteins, Figure I. Detection of protein-protein interactions between hAgo2 and hPACT or hTRBP, Figure J. hAgo2-PAZ9-mediated RNA cleavage and Figure K. hAgo2-catalyzed RNA cleavage as a function of the concentration of dsRNA-binding proteins. (PDF) [file pone.0146814.s001.pdf]

Supporting Information File

Recombinant hTRBP and hPACT modulate hAgo2-catalyzed siRNA-mediated target RNA cleavage in vitro

Sarah Willkomm[1,2], Andrea Deerberg[2], Johannes Heidemann[2], Friedemann Flügge[2], Janica Meine[2], Rui Hu[2], Rosel Kretschmer-Kazemi Far[2] and Tobias Restle[2,*]

[1]Institute of Microbiology, Single Molecule Biochemistry Lab, University of Regensburg, Regensburg, 93053, Germany and [2]Institute of Molecular Medicine, Universitätsklinikum Schleswig-Holstein, University of Lübeck, Lübeck, 23538, Germany

*To whom correspondence should be addressed. Tel: 0049-451-500-2745; Fax: 0049-451-2729; Email: restle@imm.uni-luebeck.de

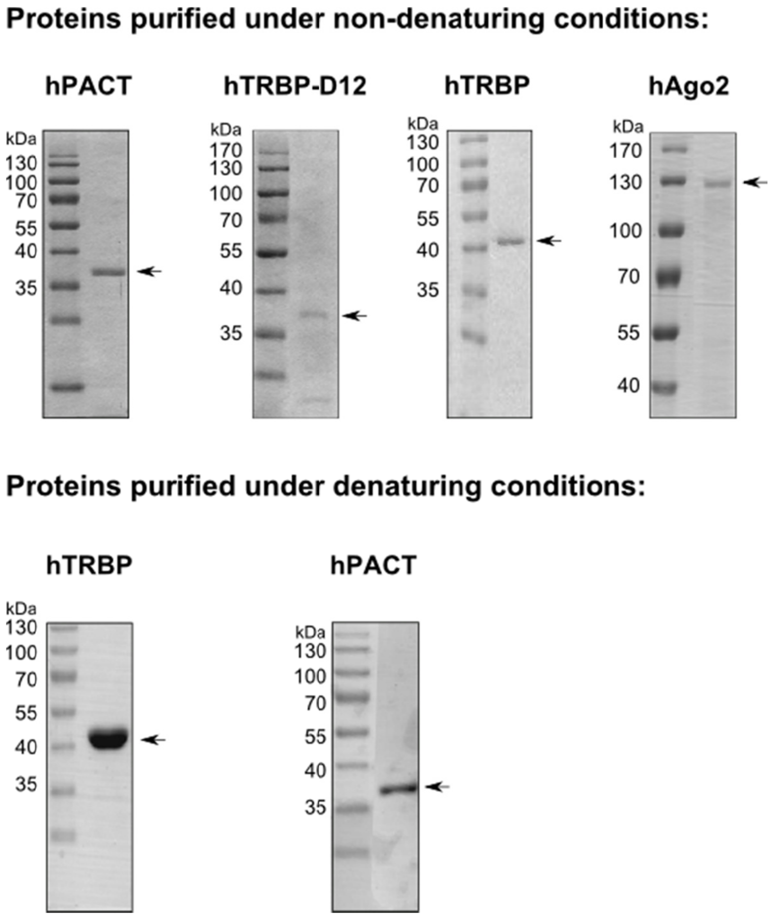

Figure A. SDS-PAGE analysis of purified recombinant proteins. Proteins were stained by Coomassie Blue. In some instances lanes for marker and protein of interest were digitally merged after electrophoresis (hTRBP-D12, hTRBP, hAgo2, hPACT^{denat}).

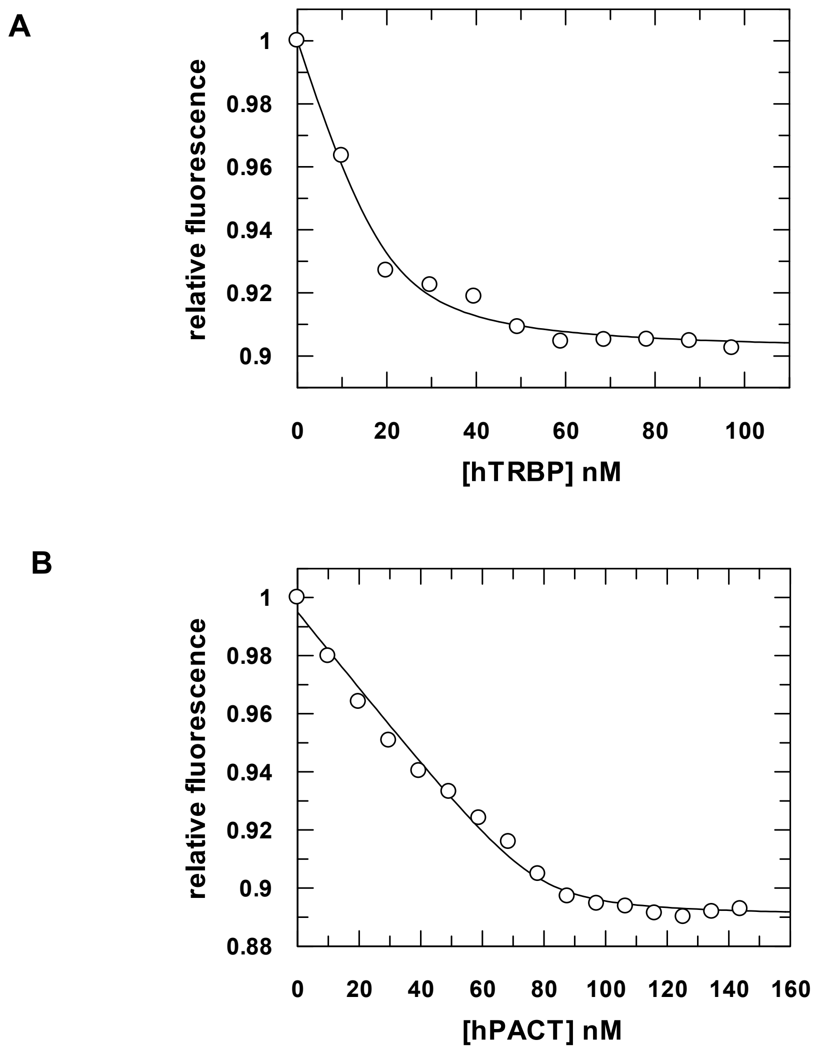

Figure B. Equilibrium titrations of hTRBP^{denat} and hPACT^{denat} with double-stranded siRNA. 30 nM fluorescently labeled ds-siRNA (aslam-FAM/slam) were titrated with increasing concentrations of hTRBP^{denat} (A) and hPACT^{denat} (B). Experimental data were fitted to a quadratic equation which yields K_d -values of 3 (± 0.7) nM and 1.4 (± 0.5) nM for hTRBP and hPACT, respectively.

A

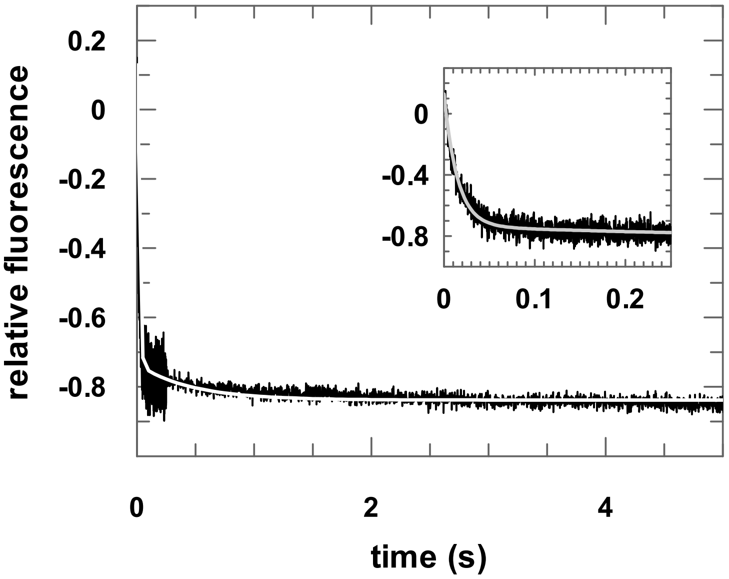

B

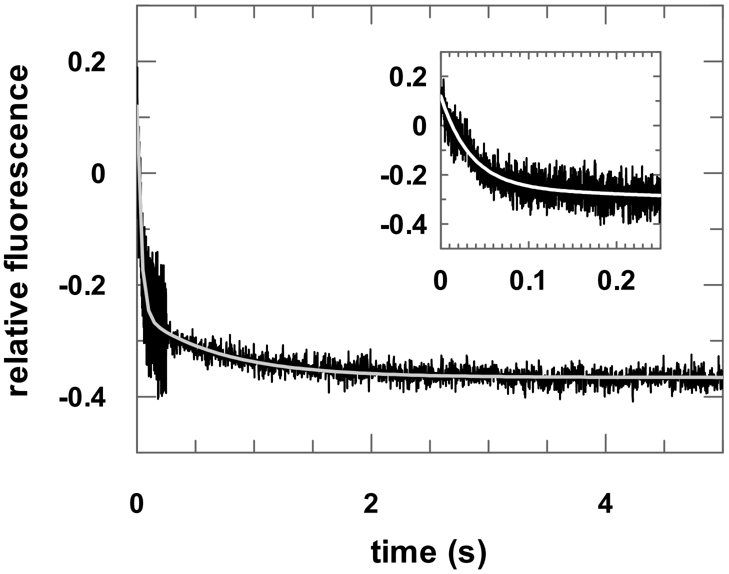

Figure C. Pre-steady state kinetic analyses of the association of hTRBP^{denat} or hPACT^{denat} and double-stranded siRNA. Representative stopped-flow graphs are shown. The inserts show the reaction on a shorter time scale. 50 nM of double-stranded siRNA carrying a 5'-FAM-label were rapidly mixed with 300 nM hTRBP^{denat} (A) or 200 nM hPACT^{denat} (B). Data were fitted to a double exponential equation yielding the following rates: k_1: 67.7 (± 0.6) s^{-1} and k_2: 2.2 (± 0.13) s^{-1} for hTRBP^{denat} and k_1: 29.5 (± 0.5) s^{-1} and k_2: 1.4 (± 0.08) s^{-1} for hPACT^{denat}.

A

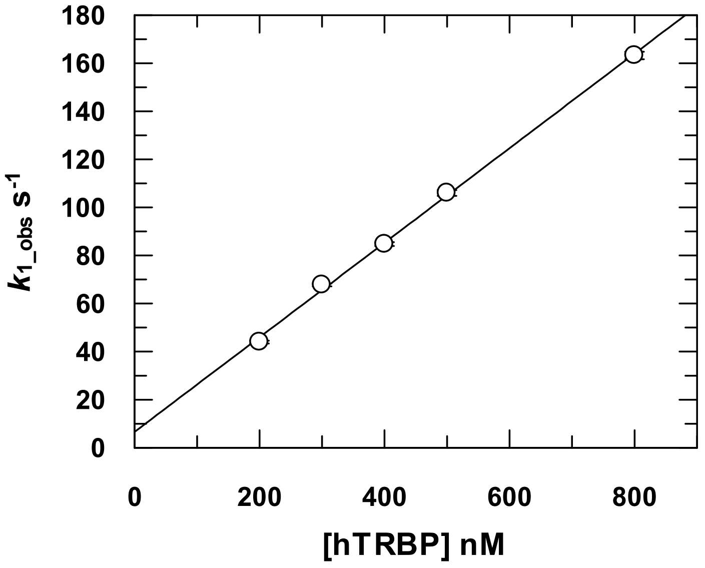

B

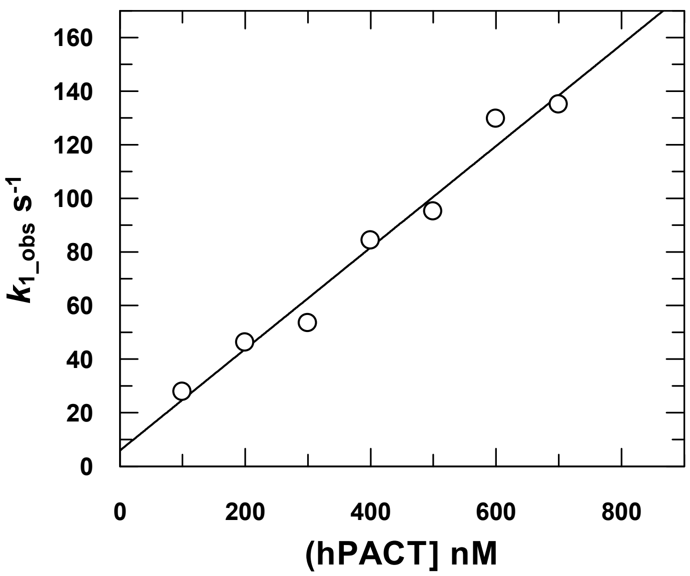

Figure D. Concentration dependencies of the first phase of double-strand siRNA binding (observed pseudo-first-order rate constant) with hTRBP^{denat} and hPACT^{denat} are shown. Increasing amounts of hTRBP^{denat} or hPACT^{denat} were mixed with 50 nM aslam-FAM/slam. k_1 was determined by the slope of the linear fit and yielded values of 2.0 (± 0.04) x 10^8 M^{-1} s^{-1} for hTRBP^{denat} and 1.9 (± 0.013) x 10^8 M^{-1} s^{-1} for hPACT^{denat}.

A

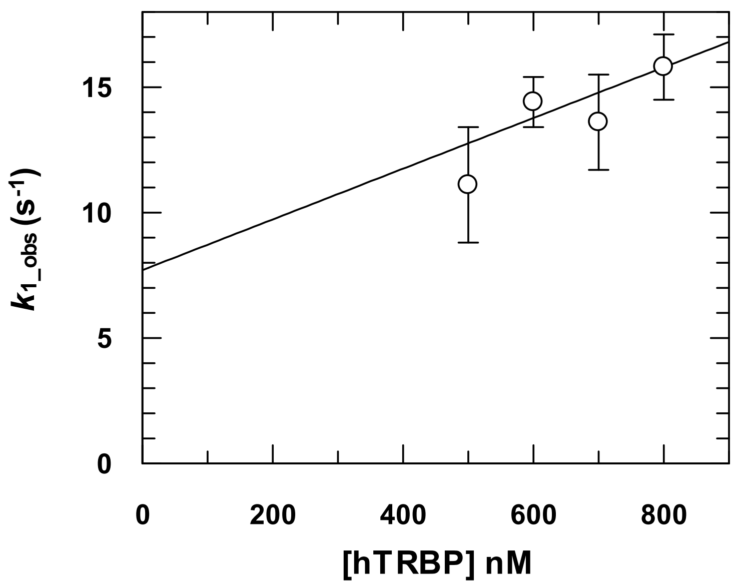

B

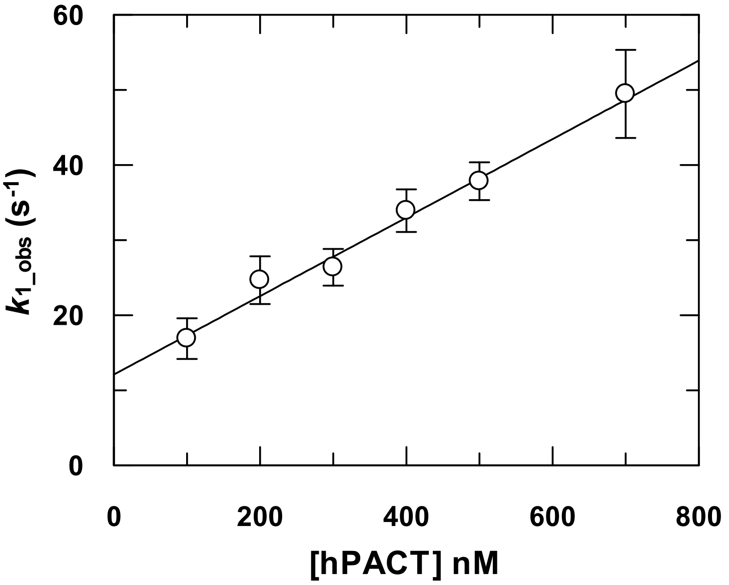

Figure E. Concentration dependencies of the first phase of double-strand siRNA binding (observed pseudo-first-order rate constant) with hTRBP^{nat} and hPACT^{nat} are shown. Increasing amounts of hTRBP^{nat} or hPACT^{nat} were mixed with 50 nM aslam-FAM/slam. k_1 was determined by the slope of the linear fit and yielded values of 0.1 (± 0.06) x 10^8 M^{-1} s^{-1} for hTRBP^{nat} and 0.5 (± 0.004) x 10^8 M^{-1} s^{-1} for hPACT^{nat}.

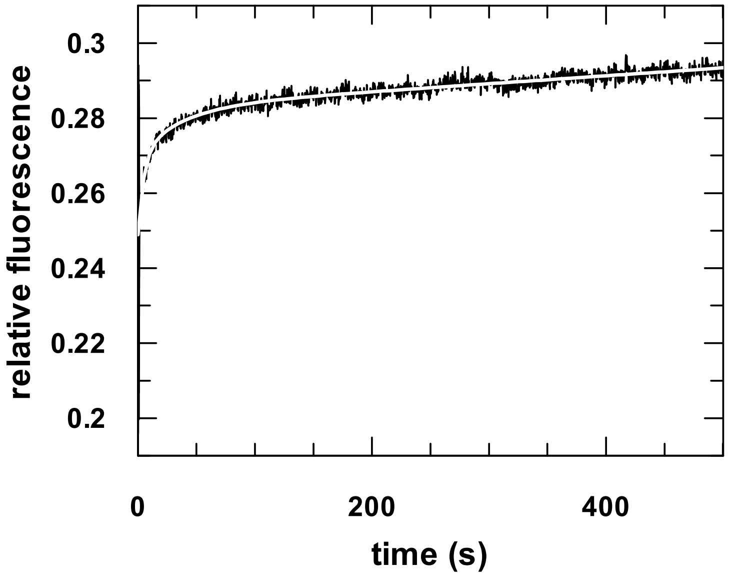

Figure F. Kinetics of the dissociation of hTRBP^{nat}/ds-siRNA-complexes. A representative stopped-flow graph is shown. Pre-assembled complexes of 1000 nM hTRBP^{nat} and 50 nM aslam-FAM/slam were rapidly mixed with 350 nM unlabeled double stranded competitor RNA. Data were fitted to an exponential equation yielding two rates k_{-1}: 0.2 (± 0.1) s^{-1} and k_{-2}: 0.03 (± 0.01) s^{-1}.

A

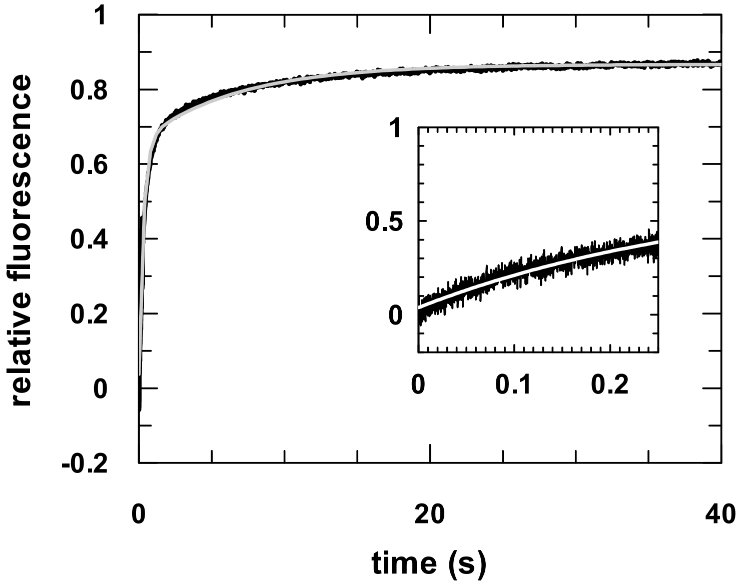

B

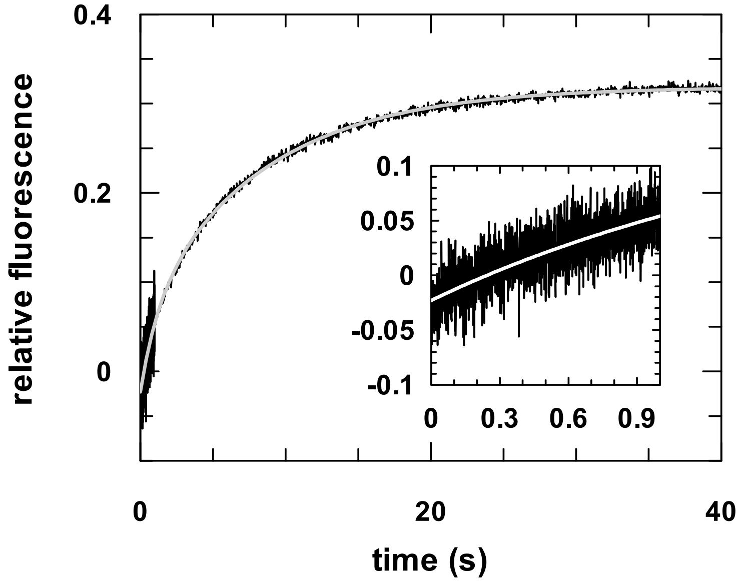

Figure G. Kinetics of the dissociation of hTRBP^{denat}/- or hPACT^{denat}/ds-siRNA-complexes. Representative stopped-flow graphs are shown. The inserts show the reaction on a shorter time scale. Pre-assembled complexes of 500 nM hTRBP^{denat} (A) or hPACT^{denat} (B) and 50 nM aslam-FAM/slam were rapidly mixed with 350 nM unlabeled double-stranded competitor RNA. Data were fitted to an exponential equation yielding two rates k_{-1}: 3.2 (± 0.03) s^{-1} and k_{-2}: 0.15 (± 0.004) s^{-1} for hTRBP^{denat} (A) and k_{-1}: 0.8 (± 0.04) s^{-1} and k_{-2}: 0.12 (± 0.002) s^{-1} for hPACT^{denat} (B).

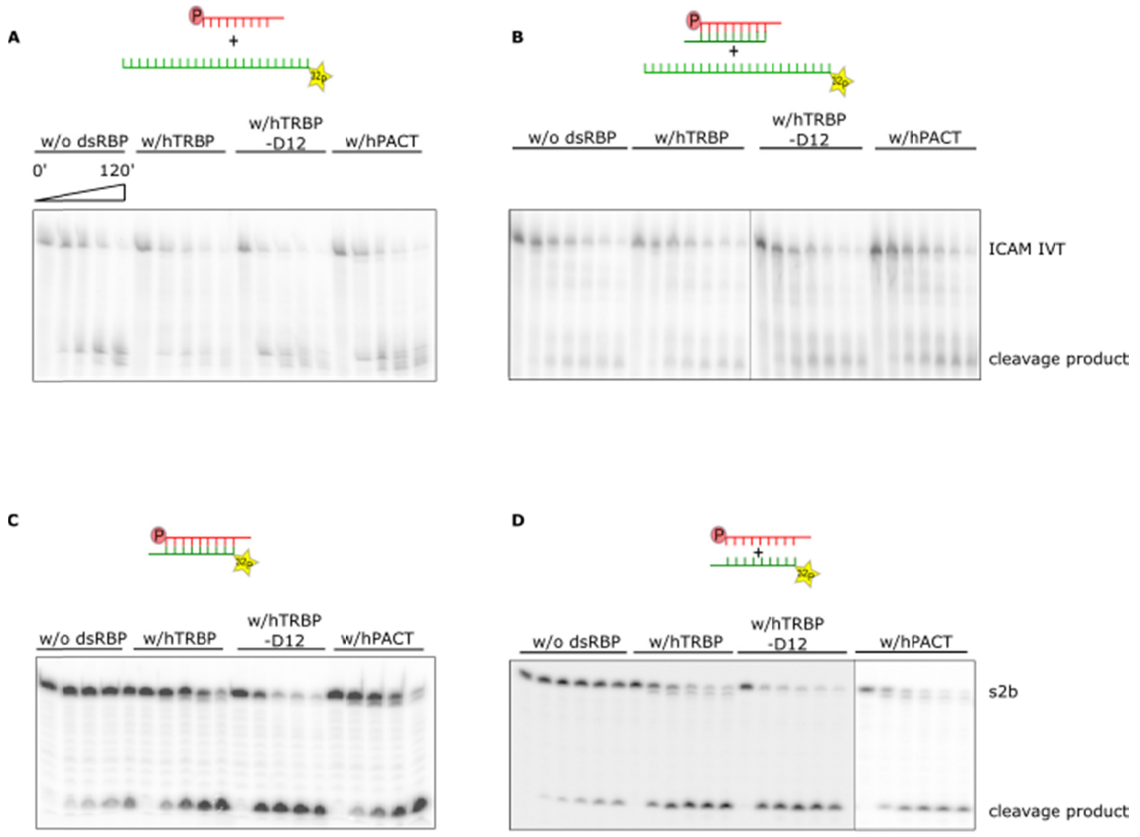

Figure H. hAgo2-mediated cleavage of target RNA in presence or absence of dsRNA-binding proteins. Cleavage assays were conducted using 2.5 µM hAgo2, 2.5 µM dsRNA-binding proteins, 100 nM guide RNA (A, D) or siRNA (B) and 2.5 nM radiolabelled target RNA (A, B, D) which is either ICAM IVT (A, B) or s2b (D) or 30 nM prehybridized siRNA duplex with radiolabeled passenger strand (C). Reactions were analyzed by 8% (w/v) (A, B) or 20% (w/v) denaturing (7 M urea) PAGE followed by autoradiography.

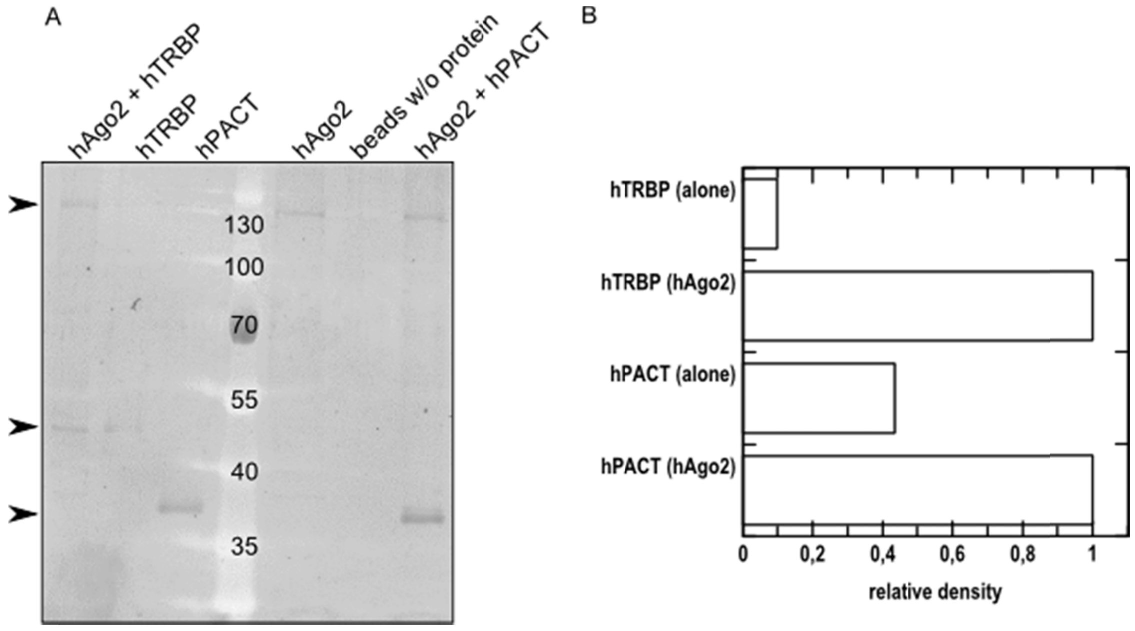

Figure I. Detection of protein-protein interactions between hAgo2 and hPACT or hTRBP. (A) DsRNA-binding proteins were incubated with hAgo2 attached to Glutathion Sepharose High Performance beads (GE Lifesciences) by its GST tag in hAgo2 binding buffer (50 mM Tris, pH 7.4, 1 mM EDTA and 10 mM DTT). After washing three times, hAgo2 elution buffer (50 mM Tris, pH 8.0, 20 mM reduced glutathione and 0.02 mM EDTA) was added to the beads. The beads were centrifuged and the proteins in the supernatant were analyzed by SDS-PAGE. Sypro® Ruby (Sigma-Aldrich)-stained protein bands were detected at a wavelength of 610 nm. The arrows indicate the position of hAgo2, hTRBP and hPACT from top to bottom. (B) Densitometric analysis of corresponding protein bands shown in part A by ImageQuant 5.2.

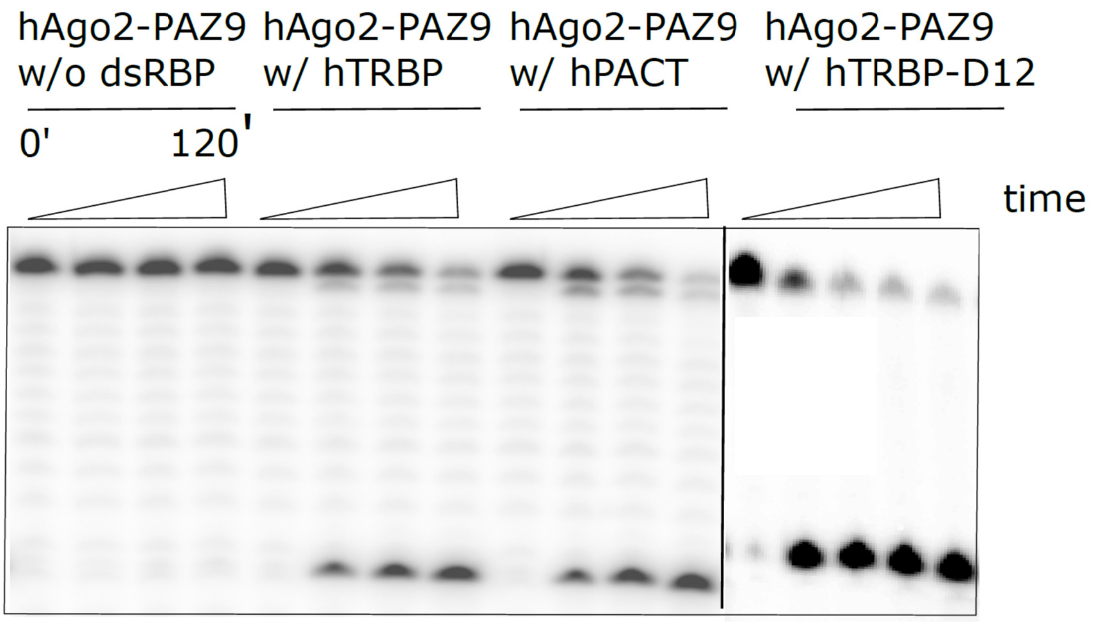

Figure J. hAgo2-PAZ9-mediated RNA cleavage. For details see Fig 6.

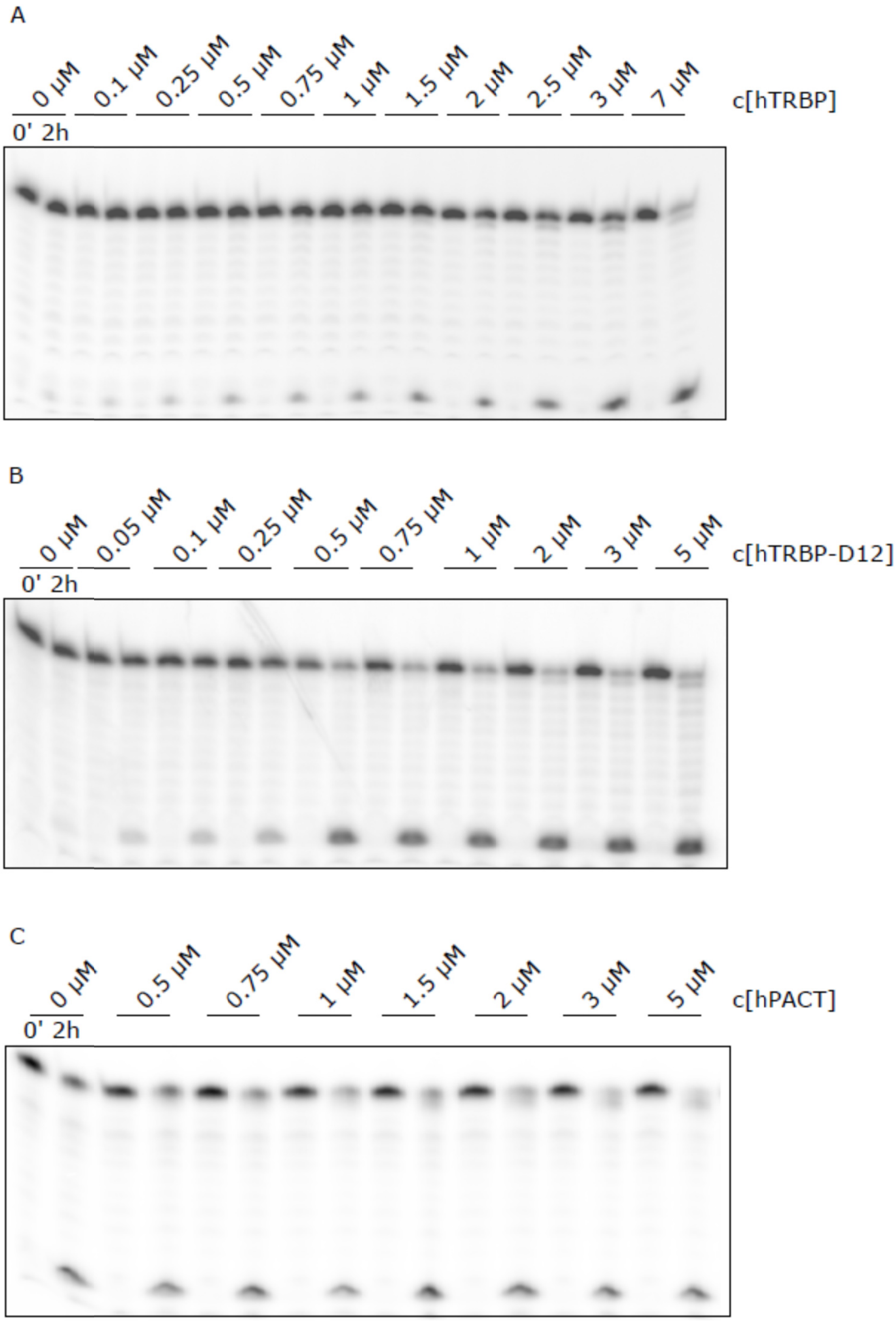

Figure K. hAgo2-catalyzed RNA cleavage as a function of the concentration of dsRNA-binding proteins. For details see Fig 7.
